# Treatment with the WNT5A-mimicking peptide Foxy-5 effectively reduces the metastatic spread of WNT5A-low prostate cancer cells in an orthotopic mouse model

**DOI:** 10.1371/journal.pone.0184418

**Published:** 2017-09-08

**Authors:** Giacomo Canesin, Susan Evans-Axelsson, Rebecka Hellsten, Agnieszka Krzyzanowska, Chandra P. Prasad, Anders Bjartell, Tommy Andersson

**Affiliations:** 1 Department of Translational Medicine, Division of Cell and Experimental Pathology, Lund University, Clinical Research Centre, Skåne University Hospital Malmö, Malmö, Sweden; 2 Department of Translational Medicine, Division of Urological Cancers, Lund University, Skåne University Hospital Malmö, Malmö, Sweden; Istituto Superiore di Sanità, ITALY

## Abstract

Prostate cancer patients with high WNT5A expression in their tumors have been shown to have more favorable prognosis than those with low WNT5A expression. This suggests that reconstitution of Wnt5a in low WNT5A-expressing tumors might be an attractive therapeutic approach. To explore this idea, we have in the present study used Foxy-5, a WNT5A mimicking peptide, to investigate its impact on primary tumor and metastasis *in vivo* and on prostate cancer cell viability, apoptosis and invasion *in vitro*. We used an *in vivo* orthotopic xenograft mouse model with metastatic luciferase-labeled WNT5A-low DU145 cells and metastatic luciferase-labeled WNT5A-high PC3prostate cancer cells. We provide here the first evidence that Foxy-5 significantly inhibits the initial metastatic dissemination of tumor cells to regional and distal lymph nodes by 90% and 75%, respectively. Importantly, this effect was seen only with the WNT5A-low DU145 cells and not with the WNT5A-high PC3 cells. The inhibiting effect in the DU145-based model occurred despite the fact that no effects were observed on primary tumor growth, apoptosis or proliferation. These findings are consistent with and supported by the *in vitro* data, where Foxy-5 specifically targets invasion without affecting apoptosis or viability of WNT5A-low prostate cancer cells. To conclude, our data indicate that the WNT5A-mimicking peptide Foxy-5, which has been recently used in a phase 1 clinical trial, is an attractive candidate for complimentary anti-metastatic treatment of prostate cancer patients with tumors exhibiting absent or low WNT5A expression.

## Introduction

Prostate cancer is the second most frequently diagnosed cancer in men and it represents one of the most common causes of cancer-related mortality in men worldwide [1,2]. Following the surgical removal of the primary tumor, the first line treatment for patients with locally advanced prostate cancer is androgen-deprivation therapy (ADT), which results in disease remission in approximately 90% of patients [3,4]. However, even if the majority of prostate cancer cells respond to ADT, androgen-insensitive tumor cell populations can still arise, and many patients develop castration-resistant prostate cancer within 2–3 years [3,5]. Although recently developed compounds such as enzalutamide (MDV3100, XTANDI^®^) and abiraterone acetate (Zytiga^®^), which specifically and efficiently inhibit androgen signaling, have demonstrated significant survival benefits for these patients, the metastatic spread of prostate cancer remains a severe clinical problem [6–8]. The cause of death in most prostate cancer patients actually results from cancer cell dissemination and the establishment of metastases in pelvic and retroperitoneal lymph nodes or in bones, but no treatments are currently available to specifically inhibit the metastatic spread of prostate cancer [9–12]. Thus, there is still a crucial need to develop novel therapies that can effectively target the metastatic dissemination of prostate cancer [13,14].

In the present study we focused on WNT5A, a non-canonical member of the Wnt family, which plays important roles in organ development, tissue orientation, cell polarity and migration [15]. Dysregulation of WNT5A has been associated with progression of various malignancies, but differences in the function of WNT5A in different types of cancer most likely reflect the fact that the cellular context is crucial in determining the action of WNT5A [16]. While WNT5A is considered to have a tumor-suppressive function in colon cancer [17], neuroblastoma [18], breast carcinomas [19], and leukemia [20], it has also been shown to promote progression of gastric cancer [21], melanoma [22], lung [23] and pancreatic cancer [24]. WNT5A expression and function have also been related to prostate cancer, but there have been conflicting reports regarding the role of this protein in the progression of this disease [25–30]. These contradictory results include reports of different *in vitro* responses to recombinant WNT5A (rWNT5A) in prostate cancer cells and different prognostic values for WNT5A expression in human prostate cancer tissue [25–30]. These conflicting data highlight the importance of directly testing the functional role of WNT5A in prostate cancer progression in appropriate animal models.

In order to restore WNT5A functions we used Foxy-5, a formylated WNT5A-derived six amino acid peptide that has been recently used in a phase 1 clinical trial in patients with advanced cancers (www.clinicalTrials.gov; NCT02020291). Foxy-5 has been characterized as a WNT5A-mimicking peptide that triggers cytosolic free calcium signaling without affecting β-catenin activation [31] and it impairs the migration and invasion of epithelial cancer cells [25,31]. Since Foxy-5 is a WNT5A mimicking peptide, one can presume that its effects will be more distinct in a cancer cell line with a low endogenous WNT5A expression. In the present study we injected WNT5A-low DU145 or WNT5A-high PC3 prostate cancer cells into the prostate of mice and then let the primary tumors establish for a period of one to three weeks before the treatment with Foxy-5 was initiated. This approach differs from our proof-of-concept study where Foxy-5 was evaluated in a breast cancer-based xenograft mouse model [32], where the treatment with Foxy-5 started simultaneously with the inoculation of cancer cells. We chose to adjust the protocol, as we believe that the approach documented in the present investigation better reflects the clinical situation. Consequently, we have used this model to resolve if Foxy-5 has an anti-metastatic effect or not. Our results show that Foxy-5 significantly reduces the early metastatic spread of WNT5A-low DU145 prostate cancer cells, but it has no effect on the metastatic spread of WNT5A-high PC3 cells. These findings indicate that Foxy-5 is a potential novel anti-metastatic compound for patients with no or low levels of endogenous WNT5A in their tumors.

## Materials and methods

### Ethics statement

All mouse experiments and experimental procedures were approved by the Regional Ethical Board, Lund University and all applicable international, national, and/or institutional guidelines for the care and use of animals were followed. All procedures performed in studies involving animals were in accordance with the ethical standards of the institution or practice at which the studies were conducted (permit number M29-13 from the Regional Ethical Board, Lund University). All surgery was performed under isoflurane anesthesia, and all efforts were made to minimize suffering. If clinical signs of illness became apparent (e.g. excessive body weight loss, hunchback or reduced motility), mice were sacrificed prior to the allowed experimental endpoint (10 weeks). In both DU145-Luc and PC3M-Luc2 experiments, it was necessary to sacrifice the animals prior to the pre-established experimental endpoint (week 9 for the DU145-Luc cells and week 6 for the PC3M-Luc2 cells).

### Cell lines and reagents

The following human prostate cancer cell lines were used: DU145 and PC-3 from American Type Culture Collection (ATCC, Manassas, VA, USA); DU145-Luciferase (DU145-Luc) from Anthem Biosciences (Bangalore, India); PC3M-Luc2 from Caliper Life Sciences (Alameda, CA). DU145, PC-3 and PC3M-Luc2 cells were cultured in RPMI-1640 medium, while DU145-Luc cells were grown in Dulbecco’s Modified Eagle Medium (DMEM). All media were supplemented with 10% heat-inactivated FBS and 1% penicillin/streptomycin. All cell lines were regularly tested to confirm the absence of mycoplasma infection. The molecular characterization of the cell lines was performed in February 2015 by LGC Standards (Cologne, Germany) and the results were then evaluated by comparison with the ATCC database (http://www.lgcstandards-atcc.org/STR_Database.aspx). Specifically, our batches of DU145-Luc revealed 94.7% match and DU145 cells revealed 100% match in comparison with ATCC standard for DU145 cells. Our batches of PC-3 and PC3M-Luc2 cells revealed 100% match in comparison with ATCC standard for PC-3 cells. The WNT5A-mimicking peptide Foxy-5 was obtained from Bachem AG (Bubendorf, Switzerland).

### *In vitro* cell viability and apoptosis assays

Cell viability assays were performed as previously described [33]. Briefly, for the cell viability assays, cells were seeded in 96-well plates (5,000 cells/well) for 24 h and were then treated with 50 μM or 100 μM Foxy-5 for an additional period of 24 h. A volume of 10 μl of MTT solution (3-(4,5-Dimethylthiazol-2-yl)-2,5-Diphenyltetrazolium Bromide) was added to each well, and the plate was incubated at 37°C for 3–4 h. The supernatant was then discarded, and 100 μl of DMSO was added to each well. After incubating the cells for 10 min at 37°C, the absorbance was measured using an ELISA plate reader at a wavelength of 450 nm.

Apoptosis assays were performed by immunofluorescence as previously described [33]. Briefly, cells were plated on coverslips in the presence of Foxy-5, vehicle or a positive control (Galiellalactone, see [33]). Cells were fixed and permeabilized as previously described [34] and stained with the M30 Cytodeath antibody (Roche Diagnostics) and 4’,6-diamidino-2-phenylindole (DAPI), according to the manufacturer’s instructions. Fluorescent images were obtained using a Nikon Eclipse 80i microscope and the NIS-Elements program.

### Invasion assays

Cell invasion was measured using BD BioCoat Matrigel Invasion Chambers (BD Biosciences, Bedford, MA) as previously described [33]. Briefly, cells were starved in Serum-Free Medium (SFM) for 24 h, harvested using Versene (Invitrogen, Carlsbad, CA), and resuspended as single cells in SFM. A total of 50,000 cells were plated in the upper transwell chamber in the presence of the indicated concentrations of vehicle or Foxy-5, and the lower chamber was filled with serum-containing medium. The cells were allowed to invade at 37°C in a humidified atmosphere of 5% CO_2_ over 24 h. After fixation with 4% paraformaldehyde, migrated cells were stained with 0.2% crystal violet, and the membranes were thoroughly washed in PBS to carefully remove any residual staining solution. The membranes were then excised, the remaining dye was solubilized using a 10% SDS solution, and the absorbance was measured at 590 nm. A variation of this invasion assay is outlined in S5c Fig. where the cells that were pre-treated with vehicle or Foxy-5 during 2 h and then centrifuged at 800 rpm for 3 minutes. Supernatants were discarded and cells were re-suspended in SFM and plated in the upper transwell chamber. Both groups of cells were then allowed to invade at 37°C in a humidified atmosphere of 5% CO_2_ over 22 h (to keep the total time at 24 h as above) in the absence of Foxy-5. Cell invasion was then studied as described above.

### RNA extraction, reverse transcriptase PCR and real-time PCR

RNA extraction, reverse transcriptase PCR and quantitative real-time PCR (qPCR) were performed as previously described [35]. Briefly, total RNA from cell lines was extracted using the RNeasy kit (Qiagen) according to the manufacturer’s instructions. Two micrograms of RNA were used for cDNA synthesis using random primers and the M-MuLV reverse transcriptase enzyme (Thermo Scientific). qPCR analysis was performed on a Stratagene Mx3005P system (Agilent Technologies) using Maxima SYBR Green/Rox according to the manufacturer’s instructions (Thermo Scientific). The relative expression of WNT5A was normalized to the expression of the TATA box binding protein (TBP) gene. The primers used were as follows: WNT5A-FW: 5’-TCAGGACCACATGCAGTA-3’, WNT5A-RV: 5’-CTCATGGCGTTCACCACC-3’; TBP-FW: 5’-GACTCTCACAACTGCACCCTTGCC-3’, TBP-RV: 5’-TTTGCAGCTGCGGTACAATCCCAG-3’.

### siRNA transfections

Transient siRNA transfections were performed with the Lipo-fectamine 2000 reagent (Invitrogen), according to the manufacturer’s instructions. The following siRNA oligonucleotides were used: from Invitrogen, anti-WNT5A siRNA #1 (s14871; 100 nM), anti-WNT5A siRNA #2 (s14872; 100 nM), Negative Control siRNA (#4390843; 100 nM) After 4 h, the transfection complex was replaced with fresh cell media supplemented with 10% FBS, and the cells were subsequently allowed to grow for 48 h prior to analysis.

### Western blotting

Protein extractions, determinations of protein concentration and western blots were performed as previously described [33]. Briefly, 40 μg of total proteins were prepared in 4x Laemmli buffer and heated to 95°C for 5 min prior to loading on an SDS-PAGE gel. After separation and transfer of the proteins to PVDF membranes, the membranes were blocked and probed with the following antibodies: anti-WNT5A (R&D Systems, 1:100); anti-α-tubulin (Santa Cruz Biotechnology, 1:10000). After washing, the membranes were incubated with either rabbit anti-goat or goat anti-rabbit/mouse HRP-conjugated secondary antibodies (Dako). Following a second wash, the separated protein bands were visualized using the Immobilon Western Chemiluminescence HRP substrate (Millipore) and were imaged and analyzed using the ChemiDoc imaging system (Bio-Rad).

### Animal experiments and *in vivo* imaging

*In vivo* experiments were performed as previously described [33]. Briefly, 1 x 10^6^ DU145-Luc or PC3M-Luc2 cells were injected into the prostate of 8-week old NMRI nude mice (Janvier Labs, Saint-Berthevin, France). Tumor growth was measured weekly using live animal bioluminescence optical imaging with the IVIS Lumina II imaging system (PerkinElmer, Hopkinton, MA, USA). Both *in vivo* experiments (DU145-Luc and PC3M-Luc2) were designed to contain 30 animals (15 per group) but 4 animals in each experiment suffered post-surgical death. When tumors in at least 50% of the injected animals were detectable by IVIS imaging, the animals were divided into two groups with similar average bioluminescence index (BLI). In the DU145-Luc experiment, the animals were grouped 3 weeks after cell injections and they were treated intraperitoneally (IP) every 2 days with either Foxy-5 (2 mg/kg) or 0.9% NaCl. Treatments were initiated 3 weeks after cell injection and lasted for 6 weeks. In the PC3M-Luc2 experiment, animals were grouped one week after cell injection and they were treated IP every 2 days with either Foxy-5 (2 mg/kg) or 0.9% NaCl. Treatments were initiated 2 weeks after cell injection and lasted for 4 weeks. The average blood volume in nude mice is approximately 2 mL. Based on this, we have calculated that each IP injection would result in 50 μM of Foxy-5 in the circulation. Since the *in vivo* IP injections were given every second day throughout the treatment period and only one dose of Foxy-5 was added at the start of each *in vitro* experiment, we believe a lower concentration of Foxy-5 for the *in vivo* experiments compared to that used *in vitro* is sufficient.

In both experiments (DU145-Luc and PC3M-Luc2) on the day of sacrifice each animal was injected with D-Luciferin (via IP) and imaged after 2 to 5 minutes under anesthesia. After the first imaging, the animal was sacrificed via overdose of anesthesia followed by cervical dislocation. Primary tumors were dissected and weighed individually; the tumor size was measured using a caliper, and the volume was calculated as previously described [36]. All organs were collected and imaged individually in order to detect the presence of metastases. The lymph nodes were collected and divided into the regional lymph nodes (RLN, in the pelvic and iliac areas up to the aortic bifurcation) and the distal lymph nodes (DLN, above the aortic bifurcation), and were analyzed separately. For each animal the whole procedure was performed within 15–20 minutes of the first D-Luciferin injection. Animals were processed one by one by the same person in order to avoid time delay and to standardize the procedure in the best possible way. All collected tissues were fixed in 10% formalin and embedded in paraffin for immunohistochemical staining. Bioluminescence data were quantified using Living Imaging software 4.2 (Xenogen Corporation). For the data shown in S2a Fig, all mice were injected subcutaneously with DU145 or DU145-Luc cells (2 x 10^6^ cells in 0.1 ml serum-free growth medium). Tumor size was measured weekly using a caliper, and the volume was calculated as previously described [36]. Mice were maintained under specific pathogen-free conditions in the research animal facility of the Clinical research Center (Malmö, Sweden). Animals had free access to food and water and were housed with a 12-hour light—dark cycle and constant temperature.

### Immunohistochemistry

Tissue sections from the primary tumors, RLN and DLN were analyzed using immunohistochemistry (IHC) to evaluate the presence of human-derived tumor cells and actively proliferating cells. Tissue sections from primary tumors were also analyzed for the presence of cleaved-caspase 3-positive cells and for the presence of CD44-positive cells. IHC staining was conducted as previously described [25] using the EnVision Flex kit and Autostainer Plus (Dako, Glostrup, Denmark), according to the manufacturer’s instructions. The following antibodies were used: anti-human vimentin (M0725, clone V9, DakoPatts, Glostrup, Denmark, 1:1000), anti-Ki67 (RM-9106, clone SP6, Thermo Scientific, Hägersten, Sweden, 1:200), anti-cleaved caspase-3 (#9661S, Rabbit Monoclonal Antibody, Cell Signaling BioNordic, Stockholm, Sweden, 1:100), anti-human CD44 (156-3C11, Thermo Scientific, CA, USA, 1:1000). For each stained slide, 20x magnification pictures were taken using the Aperio ScanScope XT Slide Scanner (Aperio Technologies, Vista, CA, USA) system for bright field microscopy. The number of positive cells per section was calculated using the Image Scope software (Aperio). The Ki67 index was calculated as the ratio between the number of Ki67-positive cells and the number of vimentin-positive cells, using the same software. CD44 membrane staining was quantified using the “membrane v1.1” algorithm within the Halo image analysis software (Indica Labs, Corrales, NM, USA). The relative number of vimentin, cleaved caspase-3, Ki67 and CD44 positive cells was calculated by dividing each value in the Foxy-5-treated group by the average value from the corresponding vehicle-treated group.

### Apoptosis detection by Tunel assay

Tissue sections from the primary tumors, RLN and DLN were analyzed for the presence of apoptotic cells using the *In Situ Cell Death Detection Kit* (Roche), according to manufacturer’s instructions. Briefly, sections were de-paraffinized and re-hydrated according to standard protocols, and subsequently permeabilized with 0,1% Triton X-100, 0,1% sodium citrate solution. After washing in PBS, sections were stained with TUNEL reaction mixture and incubated for 1 h at 37°C in the dark. Cell nuclei were stained with 4’,6-diamidino-2-phenylindole (DAPI). Sections were washed again in PBS and fluorescent images were obtained directly using a Nikon Eclipse 80i microscope and the NIS-Elements program. Positive staining was quantified using the “membrane v1.1” algorithm within the Halo image analysis software (Indica Labs, Corrales, NM, USA).

### MMP9 detection

The amount of human active Matrix Metalloproteinase 9 (MMP9) in the conditioned medium of vehicle- or Foxy-5-treated cells was measured by ELISA using the Human MMP-9 ELISA kit (#DEIA1161, Creative Diagnostics, Shirley N, USA) according to the manufacturer’s instructions.

### Statistical analyses

For the DU145-Luc *in vivo* experiment, 3 animals from the vehicle group and 2 animals from the Foxy-5 group were excluded from the study due to poor tumor growth, and the final number of animals evaluated was 10 for the vehicle treatment and 11 for the Foxy-5 treatment. For the PC3M-Luc2 *in vivo* experiment, 2 animals from the vehicle group were excluded because they were found dead during the experiment, possibly due to bladder obstruction caused by abnormal tumor growth. Therefore, the final number of animals evaluated was 11 for the vehicle treatment and 13 for the Foxy-5 treatment. All *in vitro* experiments were performed at least 3 times in triplicate or quadruplicate. All statistical analyses were performed using Graph Pad Prism software, and statistical significance was determined using ANOVA or Student's t-test. More details regarding the specific statistical analyses used for each experiment are reported in the figure legends.

## Results

**Fig 1:**
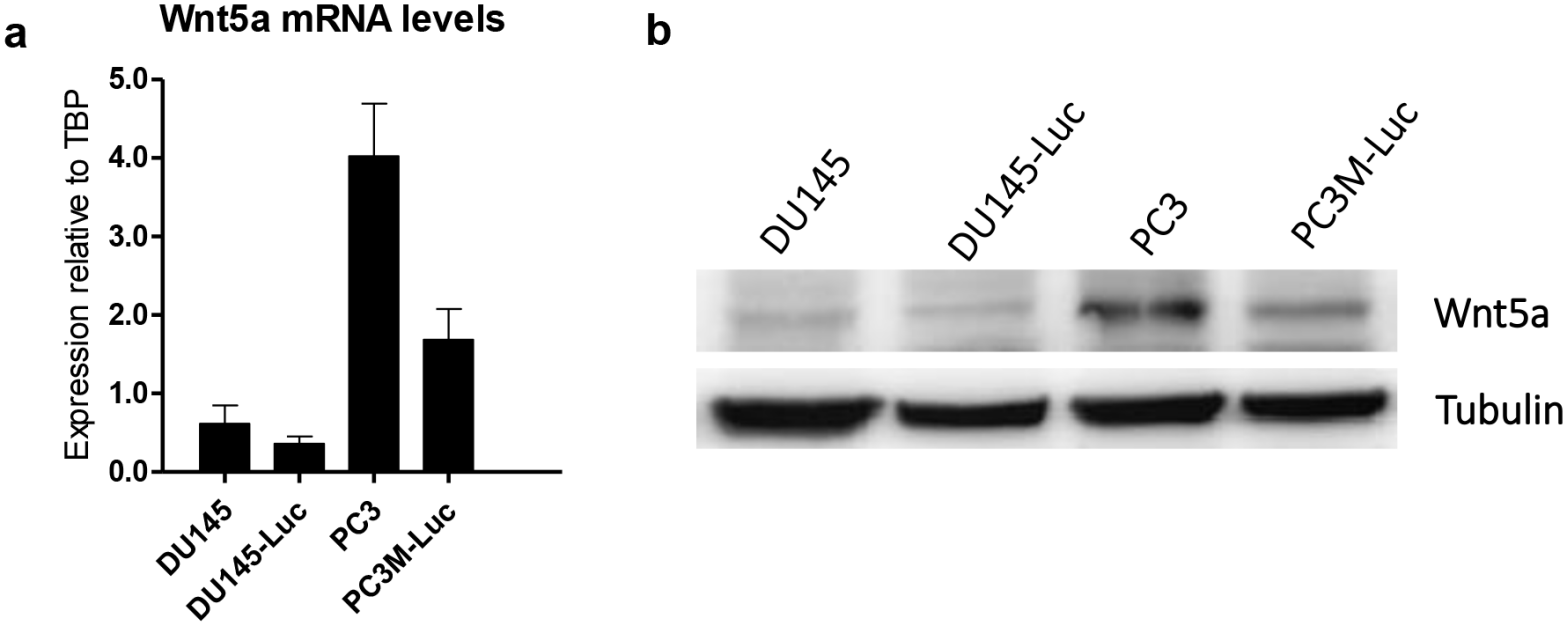
Expression of WNT5A in DU145, DU145-Luc, PC3 and PC3M-Luc2 prostate cancer cells. **(a)** qPCR analysis of the endogenous mRNA levels of WNT5A in a panel of prostate cancer cell lines (DU145, DU145-Luc, PC3, PC3M-Luc2). Results represent the mean ± s.e.m. of five independent experiments (n = 5), each of which was performed in triplicate. **(b)** Western blot analysis of the endogenous levels of WNT5A in DU145, DU145-Luc, PC3 and PC3M-Luc2 prostate cancer cell lines. A representative blot of three independent experiments (n = 3) is shown.

To establish an adequate model for studying the role of WNT5A in prostate cancer progression and in particular in metastasis, we first searched for suitable prostate cancer cell lines. According to previously published literature, we focused on DU145 and PC3 cell lines, which are known to be highly aggressive and metastatic *in vivo* [37]. Further characterization of these cells revealed that WNT5A expression is detected in DU145 and PC3 cell lines at both mRNA and protein levels; however, the level of WNT5A expression is significantly higher in PC3 cells (Fig 1).

**Fig 2:**
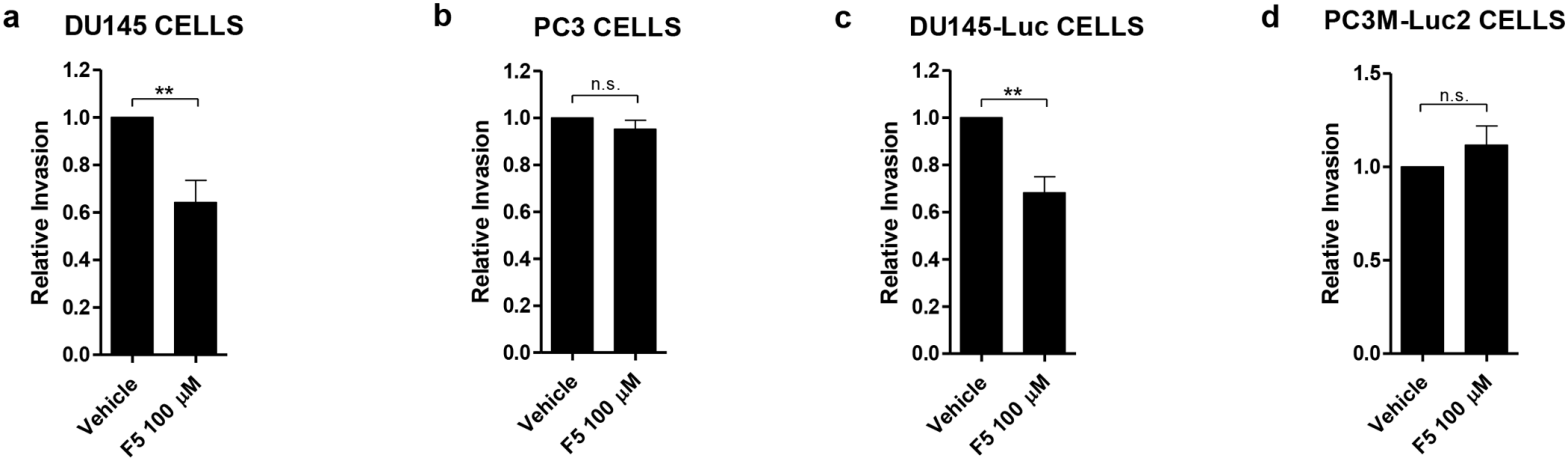
Effects of Foxy-5 on the invasion of DU145, PC3, DU145-Luc and PC3M-Luc2 cells *in vitro*. Invasion of DU145 (a), PC3 (b), DU145-Luc (c) and PC3M-Luc2 cells (d) 24 h after treatment with vehicle (0.9% NaCl) or 100 μM Foxy-5. Results represent the mean ± s.e.m. of nine (n = 9, a), six (n = 6, b and c) and four (n = 4, d) independent experiments, each of which was performed in triplicate. Statistical significance was determined using paired Student-t test with Bonferroni post hoc test (**p < 0.01, n.s. denotes not significant).

Due to this difference in endogenous WNT5A expression we decided to test both cell lines for their response to Foxy-5 in an *in vitro* invasion assay. Interestingly, we observed that Foxy-5 treatment significantly reduced DU145 cell invasion by 40% (Fig 2a), but it had no effect on PC3 cell invasion (Fig 2b). These data are in accordance with previous results showing that rWNT5A impairs DU145 cell invasion whereas it has no effect on PC3 cell invasion [25]. To be able to visualize the continuous growth and dissemination of prostate cancer cells *in vivo*, it is advantageous to use luciferase-expressing cells, but it is well known that the transfection of cancer cell lines can affect their behavior. This led us to perform experiments to ascertain that the DU145-Luc and PC3M-Luc2 cells, intended for the *in vivo* experiments, did not differ in their response to Foxy-5 from the parental DU145 and PC3 cells. Our results show that Foxy-5 reduces DU145-Luc cell invasion by 40%, while it has no effect on the invasion of PC3M-Luc2 cells (Fig 2c and 2d). This indicates that the *Luc* transfection of DU145 and PC3 cells does not affect their responsiveness to Foxy-5 *in vitro*.

Next, we analyzed the effects of Foxy-5 on parental DU145 cell viability by MTT assay and we found that Foxy-5 stimulation for 24 h led to no significant effects at either 50 μM or 100 μM (S1a Fig). A similar lack of response was also observed in DU145-Luc cells (S1b Fig). Moreover, when we performed the immunofluorescence staining with the M30-Cytodeath antibody, we did not detect any apoptosis in DU145-Luc cells after 24 h of treatment with either vehicle or Foxy-5 (S1c Fig). In addition to the above results, we also found that parental DU145 and DU145-Luc cells exhibit similar growth in a subcutaneous *in vivo* model (S2a Fig). These results support the use of DU145-Luc cells for our *in vivo* studies.

**Fig 3:**
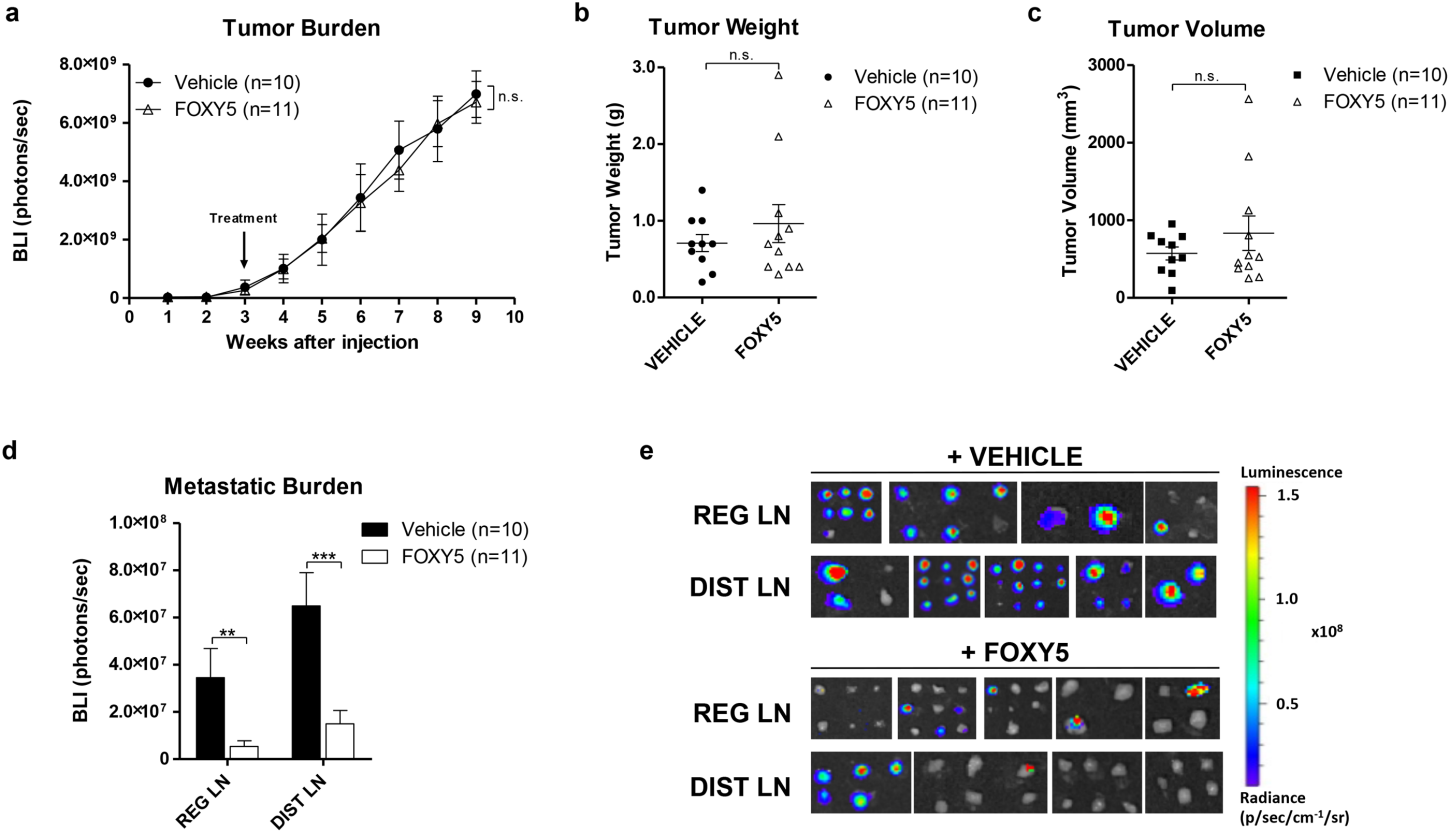
Foxy-5 reduces DU145-Luc metastatic spread *in vivo* without affecting primary tumor growth. Animals were administered either vehicle (0.9% NaCl) or Foxy-5 (2 mg/kg in 0.9% NaCl) by intraperitoneal injections every other day between weeks 3 and 9. **(a)** Tumor growth curve of vehicle- and Foxy-5-treated animals represented as BLI (Bio-Luminescence Index). The BLI was measured weekly for each animal by quantifying the total photon flux emitted by the tumor. Results are presented as the mean ± s.e.m.; statistical significance was determined using two-way ANOVA with Bonferroni post hoc test (n.s. denotes not significant). **(b)** Primary tumor weight of vehicle- and Foxy-5-treated animals. Mice were sacrificed, and primary tumors were excised and weighed individually. Results are presented as the mean ± s.e.m.; statistical significance was determined using unpaired Student-t test (n.s. denotes not significant). **(c)** The primary tumor volume of vehicle- and Foxy-5-treated animals was measured with a caliper at the time of necropsy. Results are presented as the mean ± s.e.m.; statistical significance was determined using unpaired Student-t test (n.s. denotes not significant). **(d)** Quantification of the total photon flux emitted by regional and distal lymph nodes of vehicle- and Foxy-5-treated animals. Results are presented as the mean ± s.e.m.; statistical significance was determined using unpaired Student-t test (**p < 0.01, ***p < 0.001, n.s. denotes not significant). One to six lymph nodes per animal in each group (vehicle- and Foxy-5-treated) were included in the analysis. **(e)** Representative bioluminescence images of metastatic lymph nodes from vehicle- and Foxy-5-treated animals. Images were taken directly after each animal had been sacrificed. One to six lymph nodes per animal in each group (vehicle- and Foxy-5-treated) were included in the analysis.

**Fig 4:**
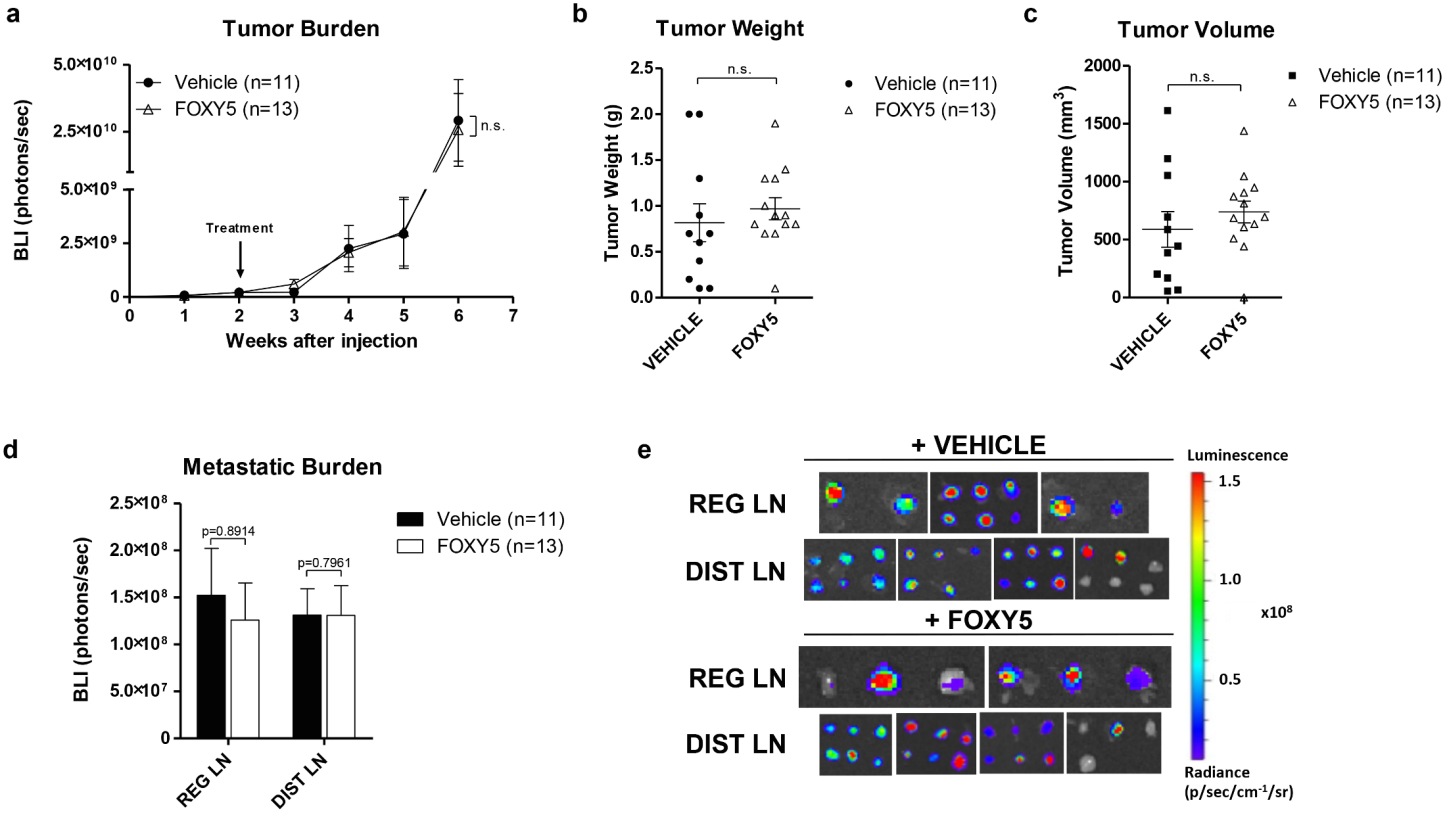
Foxy-5 doesn’t affect the *in vivo* metastatic spread or the primary tumor growth of PC3M-Luc2 cells. Animals were administered either vehicle (0.9% NaCl) or Foxy-5 (2 mg/kg in 0.9% NaCl) by intraperitoneal injections every other day between weeks 2 and 6. **(a)** Tumor growth curve of vehicle- and Foxy-5-treated animals represented as BLI (Bio-Luminescence Index). The BLI was measured weekly for each animal by quantifying the total photon flux emitted by the tumor. Results are presented as the mean ± s.e.m.; statistical significance was determined using two-way ANOVA with Bonferroni post hoc test (n.s. denotes not significant). **(b)** Primary tumor weight of vehicle- and Foxy-5-treated animals. Mice were sacrificed, and primary tumors were excised, measured and weighed individually. Results are presented as the mean ± s.e.m.; statistical significance was determined using unpaired Student-t test (n.s. denotes not significant). **(c)** The primary tumor volume of vehicle- and Foxy-5-treated animals was measured with a caliper at the time of necropsy. Results are presented as the mean ± s.e.m.; statistical significance was determined using unpaired Student-t test (n.s. denotes not significant). **(d)** Quantification of the total photon flux emitted by regional and distal lymph nodes of vehicle- and Foxy-5-treated animals. Results are presented as the mean ± s.e.m.; statistical significance was determined using unpaired Student-t test. One to six lymph nodes per animal in each group (vehicle- and Foxy-5-treated) were included in the analysis. **(e)** Representative bioluminescence images of metastatic lymph nodes from vehicle- and Foxy-5-treated animals. Images were taken directly after each animal had been sacrificed. One to 6 lymph nodes per animal in each group (vehicle- and Foxy-5-treated) were included in the analysis.

In order to study the effects of Foxy-5 on tumor growth and metastatic spreading *in vivo*, immunodeficient NMRI nude mice were injected orthotopically with cells expressing low levels of WNT5A (DU145-Luc) or with cells expressing higher levels of WNT5A (PC3M-Luc2). Tumors were allowed to establish during a time period of 1 to 3 weeks. Once detectable in at least 50% of the injected animals via BLI, treatment began with either vehicle or Foxy-5. In both DU145-Luc and PC3M-Luc2 experiments, the bioluminescence analysis of tumor growth (BLI signal) revealed no significant differences between the vehicle-treated and the Foxy-5- groups (Figs 3a and 4a, S2c and S3a Figs). There was no significant difference in animal weight between the two groups, indicating that the treatment with Foxy-5 was well tolerated (S2b and S3b Figs). Moreover, we observed no differences in tumor weight (Figs 3b and 4b) or tumor volume (Figs 3c and 4c) between vehicle- and Foxy-5 treated groups in neither DU145-Luc nor PC3M-Luc2 inoculated animals. Necropsies of the mice revealed the presence of metastases in different organs, as detected by *in vivo* bioluminescence assay and showed in S1 and S2 Tables. However, the low incidence of metastasis to spleen, bones, lungs and liver prevented us from performing further analyses on these tissues, thus we decided to focus our study on the initial metastatic spread to the lymph nodes. Importantly, the quantification of the BLI signal in the RLN and DLN of animals injected with DU145-Luc cells showed that Foxy-5 significantly reduced metastases to both RLN by 90% and to DLN by 75% (Fig 3d and 3e). On the contrary, the same quantification in the lymph nodes of animals injected with PC3M-Luc2 cells showed no difference between the vehicle- and the Foxy-5-treated groups (Fig 4d and 4e).

**Fig 5:**
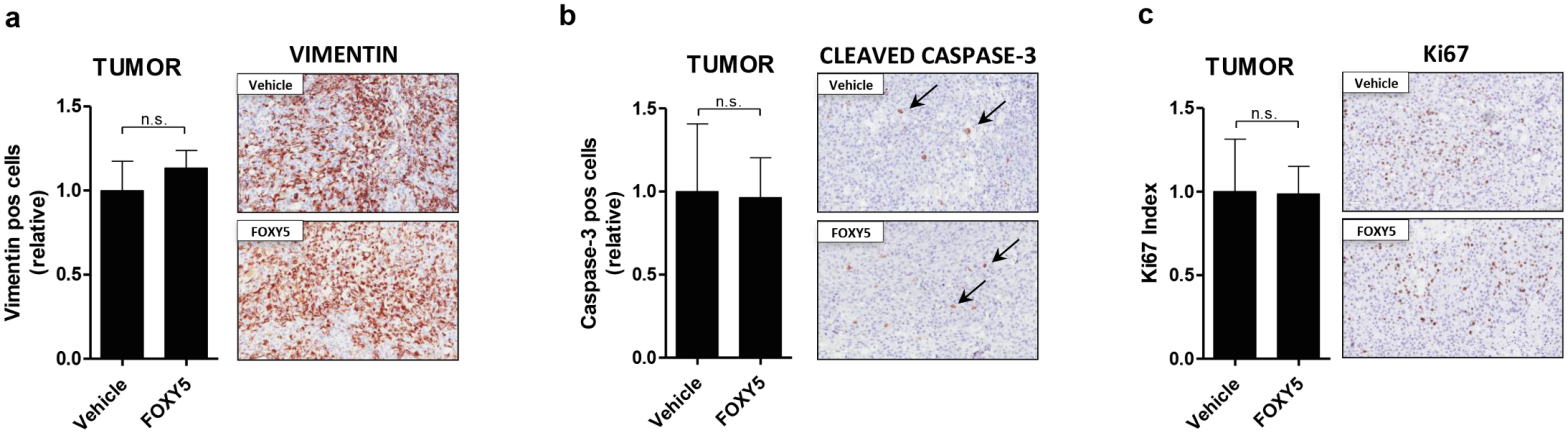
Foxy-5 does not affect the apoptosis or proliferation of DU145-Luc tumor cells *in vivo*. **(a)** Relative number of vimentin-positive cells and representative images of vimentin-positive areas in primary tumors of vehicle- and Foxy-5-treated animals. **(b)** Relative number of cleaved-caspase 3-positive cells and representative images of cleaved-caspase 3-positive areas in primary tumors of vehicle- and Foxy-5-treated animals. **(c)** Ki-67 index and representative images of Ki-67-positive areas in primary tumors of vehicle- and Foxy-5-treated animals. All results are presented as the mean ± s.e.m.; statistical significance was determined using unpaired Student-t test (n.s. denotes not significant). Images were taken with a 20X objective (Scale bar = 100 μm).

To complement the above data showing an anti-metastatic effect of Foxy-5 in animals inoculated with DU145-Luc cells, we stained their primary tumors, RLN and DLN with specific antibodies to identify tumor cells (human-vimentin staining), apoptotic cells (cleaved-caspase 3 staining) or actively proliferating cells (Ki67 staining). Additionally, apoptotic cells were also visualized by Tunel assay in tumors, RNL and DLN. Immunohistochemical analyses of the primary tumors revealed no significant differences in the number of vimentin-positive cells (Fig 5a), apoptotic cells (Fig 5b) or proliferating cells (Fig 5c), which is consistent with the BLI data (Fig 3a).

**Fig 6:**
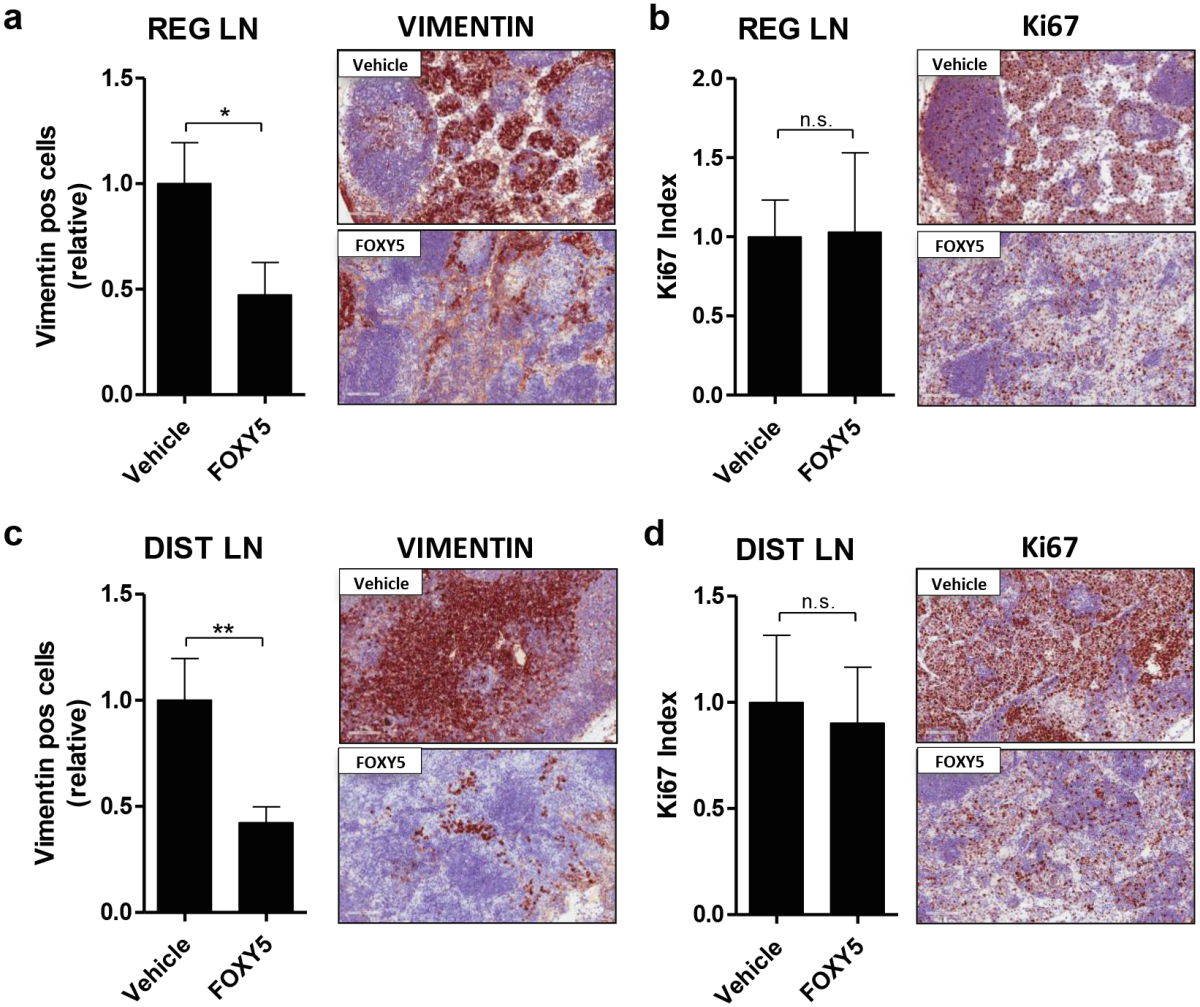
Foxy-5 reduces the number of tumor cells in lymph nodes without affecting tumor cell proliferation. **(a, c)** Relative number of vimentin-positive cells and representative images of vimentin-positive areas in (a) regional lymph nodes and (c) distal lymph nodes from vehicle- and Foxy-5-treated animals. **(b, d)** Ki-67 index and representative images of Ki-67-positive areas in (B) regional lymph nodes and (d) distal lymph nodes from vehicle- and Foxy-5-treated animals. Results are presented as the mean ± s.e.m.; statistical significance was determined using unpaired Student-t test (*p < 0.05, **p < 0.01, n.s. denotes not significant). Images were taken with a 20X objective (Scale bar = 100 μm).

The reduced vimentin staining of RLN and DLN in the Foxy-5 treated animals (Fig 6a and 6c) confirmed the decreased BLI intensities observed in the lymph nodes *in vivo* (Fig 3d and 3e), suggesting that Foxy-5 significantly reduces the number of tumor cells in the RLN and DLN. In accordance with the *in vitro* and the *in vivo* data from the primary tumors, Foxy-5 had no significant effects on the number of proliferating cells in the RLN and DLN (Fig 6b and 6d). Moreover, the quantification of Tunel-positive cells in tumors, RLN and DLN showed no difference between vehicle- and Foxy-5 treated animals, which indicates that Foxy-5 doesn’t have any effect on apoptosis in vivo (S4 Fig). Taken together, our results show that Foxy-5 specifically inhibits the early metastatic spread of WNT5A-low (DU145-Luc) but not WNT5A-high (PC3M-Luc2) cells. This effect of Foxy-5 on DU145-Luc prostate cancer cells occurred without affecting the growth of the primary tumor or the proliferation of tumor cells.

Since WNT5A inhibits breast cancer cell migration and invasion in part by reducing the levels of CD44 [38,39], we have explored if WNT5A signaling regulates the expression of this protein also in prostate cancer tissue. This analysis was performed on all DU145-Luc primary tumors treated with vehicle or Foxy-5. The results obtained show no differences in the expression levels of CD44 between the vehicle- and Foxy-5-treated groups (S5a Fig). Additionally, we also investigated the possibility that WNT5A signaling caused a reduced secretion of MMP9 in prostate cancer cells. The rationale behind this is that WNT5A signaling is known to impair invasion of breast cancer cells in part by reducing extracellular MMP9 activity [40]. However, we didn’t find any significant reduction in the secretion of MMP9 after Foxy-5 treatment from neither DU145 nor PC3 cells (S5b Fig). These data indicate that neither alteration in CD44 expression nor MMP9 secretion are part of the mechanism whereby Foxy-5 exerts its anti-invasion and anti-metastatic effects on DU145 prostate cancer cells. Since the half-life of peptides *in vivo* is known to be very short, we tested if a brief exposure to Foxy-5 would have effect on DU145 cell invasion. For this, we pre-treated DU145 cells with vehicle or Foxy-5 for 2 h and then we plated the cells for the invasion assay in the absence of Foxy-5. Interestingly, we found that a 2 h exposure to Foxy-5 is sufficient enough to significantly reduce the invasion of DU145 cells *in vitro* (S5c Fig).

## Discussion

It has previously been shown that preserved expression of WNT5A in the primary tumor is associated with increased time to biochemical recurrence in patients with low-grade prostate cancer [26]. Moreover, patients with high endogenous WNT5A levels show a longer survival time and an overall better outcome compared to patients with low WNT5A levels [25]. Consequently, the group of prostate cancer patients that would most highly benefit from the reconstitution of WNT5A functions is the one that includes patients with absent or low WNT5A expression in their tumors. These indications prompted us to test the effect of the WNT5A agonist Foxy-5 on the invasion of both DU145 (with a low endogenous expression of WNT5A) and PC3 prostate cancer cells (with high endogenous level of WNT5A). As expected, our data clearly show a significant effect of Foxy-5 on invasion of WNT5A-low DU145 cells but not on WNT5A-high PC3 cells. Furthermore, our results reveal that although Foxy-5 impairs DU145 cell invasion it does not affect viability or apoptosis *in vitro*. Our *in vivo* data clearly show that animals inoculated with WNT5A-low DU145 cells and treated with Foxy-5 have a significantly reduced metastatic spread to regional and distal lymph nodes. In good agreement with our *in vitro* data, Foxy-5 does not significantly affect apoptosis or proliferation of tumors derived from DU145 cells *in vivo*. In accordance with our finding that Foxy-5 did not affect *in vitro* invasion of PC3 cells, it did not impair the metastatic spread of the WNT5A-high PC3 cells *in vivo*. If we combine our *in vitro* and *in vivo* data, we can then conclude that the mechanism behind the observed effects of Foxy-5 on tumor metastases *in vivo* is most readily explained by impaired DU145 cell migration and invasion.

There are several intracellular signaling pathways and adhesion molecules through which WNT5A has been shown to regulate migration and invasion of tumor cells. In breast cancer, for example, WNT5A is known to inhibit cell migration and invasion partly by reducing the levels of CD44 [38]. Specifically, it has been shown that the WNT5A-mediated suppression of CD44 reduces the downstream AKT signaling, which further explains how WNT5A signaling impairs breast cancer cell migration and invasion [39]. CD44 is known to play a similar role in prostate cancer, as it mediates the adhesion between prostate cancer cells and endothelial cells and it is also implicated in prostate cancer cell invasion [41,42]. However, we found no effects of Foxy-5 on the expression of CD44 *in vivo* and therefore we conclude that the anti-metastatic effect of Foxy-5 observed in DU145 cells is not mediated via CD44.

It has also been shown that WNT5A impairs breast cancer cells invasion partly by reducing MMP9 activity [40]. However, we didn’t find any significant reduction in the secretion of active MMP9 after the treatment with Foxy-5 in either DU145 or PC3 prostate cancer cells. Despite the fact that MMPs are key regulators of invasion in prostate cancer cells [43–45], our results indicate that MMP9 is not a target of Foxy-5 in DU145 prostate cancer cells, which is in contrast to breast cancer cells [40]. Importantly, our present results lend direct support to recent findings implicating WNT5A in the suppression of prostate cancer metastases through a complex network of signaling proteins, including Snail, Cyclin D1 and c-Myc [46]. Moreover, WNT5A may be involved in the regulation of prostate cancer cells adhesion, as already shown in several other tumors [31,47–49]. Thus, we conclude that WNT5A signaling impairs prostate and breast cancer metastases differently and in each case via several parallel mechanisms.

Although we believe that the difference in response to Foxy-5 between DU145 and PC3 cells is related to their endogenous expression of WNT5A, an alternative explanation could be a difference in WNT5A receptor expression. WNT5A is known to elicit non-canonical signaling upon binding to different receptor/co-receptor complexes, including ROR-2 and Frizzled receptors [50,51]. Both DU145 and PC3 cell lines express ROR-2 [46] and it is known that the WNT5A associated Frizzled receptors Fzd-2, Fzd-3, Fzd-5 and Fzd-7 are also expressed on these cells as well as on other PCa cell lines [46,52–54]. In addition, the Fzd-5 receptor, a commonly described receptor for WNT5A, has an even higher expression (at both mRNA and protein level) in PC3 cells compared with DU145 cells [55]. For these reasons, we believe that a difference in the expression of Frizzled or ROR-2 receptors is an unlikely explanation of our opposite findings in DU145 and PC3 cells.

The use of Foxy-5 to reconstitute WNT5A functions has already been proven effective in reducing spontaneous metastases to the lungs and liver without affecting primary tumor growth in a breast cancer mouse model [32]. Of note, there is an important difference between the present study and our previous study, where we demonstrated anti-metastatic effect of Foxy-5 in breast cancer-based xenograft models. While in the previous study the Foxy-5 treatment regimen was started simultaneously with the inoculation of the breast cancer cells [32], in the present study we started the Foxy-5 treatment three weeks after the inoculation of the tumor cells, which reflects a more clinical relevant situation. Preliminary unpublished data indicate that the half-life of Foxy-5 in the blood circulation is around 30 minutes in rats. This short half time may indicate that Foxy-5 undergoes the so-called *hit-and-run* effect, typical of small peptides in the blood circulation. Compared to a long stimulation, the *hit-and-run* effect indicates that this peptide binds quickly to its receptor, and that this quick binding is sufficient to guarantee a stable effect on the tumor cells. In agreement with this, we found that a short *in vitro* exposure to Foxy-5 (2 hours) is enough to induce a significant reduction in DU145 cell invasion, supporting the idea that Foxy-5 has a quick mode of action. However, it should be noted that while this *in vitro* effect was seen after a single stimulation with Foxy-5, the *in vivo* experiments were analyzed after continuous Foxy-5 stimulations every second day. Moreover, the *hit-and-run* effect also implies less toxicity, which is indeed a benefit. Our results show that Foxy-5 specifically targets cell invasion and early metastatic spread without affecting cell viability, apoptosis or proliferation both *in vitro* and *in vivo*. These results are thus in good agreement with our previously published data on clinical tumor material [25,26] and they demonstrate that treatment with Foxy-5 is an attractive anti-metastatic therapeutic approach, even after a primary tumor has been established.

As previously mentioned, there have been conflicting reports regarding the role of WNT5A in the progression of prostate cancer [25–29]. One study examining a small cohort of prostate cancer patients indicated a worse outcome for patients with high endogenous WNT5A levels in their cancer tissues [27]. A second more recent study reported the association of the non-canonical Wnt pathway with biochemical recurrence and metastasis in aggressive prostate cancer [29]. Another recent publication implicates non-canonical Wnt signaling in prostate cancer progression and anti-androgen resistance [30]. However, the main limitation of the three cited studies is the small size of the analyzed cohorts (98, 40 and 13 patients, respectively), which indicates that this association is probably limited to a small subgroup of prostate cancer patients. Our present *in vitro* and *in vivo* data are highly consistent with the findings reported in three other studies examining larger cohorts of prostate cancer patients and showing that patients with high endogenous WNT5A protein levels in their tumors have better outcomes and longer overall survival times compared with patients with low WNT5A levels [25,26,28].

Thus, our study further supports the use of Foxy-5 as a future treatment for prostate cancer patients that lack or show reduced endogenous expression of WNT5A in their primary tumor. According to our results, such a treatment would delay or inhibit the initial metastatic spread and thus the establishment of local and distal metastases in these patients. Moreover, the fact that Foxy-5 selectively targets metastatic dissemination is very interesting from a therapeutic perspective, since it opens the possibility for combined treatments of prostate cancer patients. As previously mentioned, important progress has been made in the treatment of prostate cancer [6–8,56]; yet further improvements can be achieved by combining different therapeutic regimens [57,58]. Since Foxy-5 exhibits a unique anti-metastatic effect and since no toxic effects were detected in a recently completed phase 1 trial (www.clinicalTrials.gov; NCT02020291), we hypothesize that this peptide can be used in combination with presently used cytotoxic compounds and thus represents a new strategy for the treatment of prostate cancer patients with low expression of WNT5A. Through such a combined approach, one might accomplish a more efficient reduction in the formation and growth of metastatic foci, resulting in an overall better clinical outcome and in a substantial improvement of quality of life in this group of patients.

## Conclusion

In conclusion, this study shows that the WNT5A-mimicking peptide Foxy-5 significantly reduces the early metastatic spread of WNT5A-low DU145 prostate cancer cells in an *in vivo* orthotopic xenograft mouse model. These results indicate that the Foxy-5 small peptide is an attractive complementary candidate for establishing a novel anti-metastatic treatment for prostate cancer patients with no or low WNT5A expression in their tumors.

## Supporting information

S1 Fig Effect of Foxy-5 on viability and apoptosis *in vitro*. **(a-b)** MTT viability assays on DU145 (a) and DU145-Luc cells (b) after 24 h of treatment with vehicle (0.9% NaCl) or 50/100 μM Foxy-5. Results represent the mean ± s.e.m. of seven (n = 7) and eleven (n = 11) independent experiments, respectively, each of which was performed in quadruplicate. Statistical significance was determined using one-way ANOVA with Bonferroni post hoc test (n.s. denotes not significant). **(c)** Immunofluorescence detection of apoptotic DU145-Luc cells after 24 h of treatment with vehicle (left panels), 100 μM Foxy-5 (middle panels) or 5 μM of the apoptosis inducing compound Galiellalactone (right panels). Apoptotic cells were visualized with the M30-cytodeath antibody, and all nuclei were stained with DAPI. Images were taken with a 10X objective (Scale bar = 50 μm). The indicated areas in the middle panels are magnified and shown in the three lower panels.
(TIF)

S2 Fig *In vivo* growth of DU145 and DU145-Luc tumors. **(a)** A comparison of tumor growth between DU145 and DU145-Luc cells injected subcutaneously into the flanks of NMRI nude mice (n = 10 per group). Tumor size was measured weekly with a caliper, and the volume was calculated as previously described [36]. Results are presented as the mean ± s.e.m.; and statistical significance was determined using two-way ANOVA with Bonferroni post hoc test (n.s. denotes not significant). **(b)** Average weight of NMRI nude mice orthotopically injected with DU145-Luc cells and treated with vehicle (0.9% NaCl) or 2 mg/kg Foxy-5, as previously described. Mice were weighed twice per week starting the first week after inoculation of the cells until the end of the treatment period (week 9). Results are presented as the mean ± s.e.m.; and statistical significance was determined using two-way ANOVA with Bonferroni post hoc test (n.s. denotes not significant). **(c)** Representative bioluminescence images of NMRI nude mice orthotopically injected with DU145-Luc cells and treated via intraperitoneal injections with either vehicle (NaCl 0.9%) or Foxy-5 (2 mg/kg in 0.9% NaCl) every other day between weeks 3 and 9. Images were taken weekly starting 1 week after the inoculation of the cells until the end of the treatment period.
(TIF)

S3 Fig Effect of Foxy-5 on PC3M-Luc2 cell i*n vivo*. **(a)** Representative bioluminescence images of NMRI nude mice orthotopically injected with PC3M-Luc2 cells and treated via intraperitoneal injections with either vehicle (NaCl 0.9%) or Foxy-5 (2 mg/kg in 0.9% NaCl) every other day between weeks 2 and 6. Images were taken weekly starting 1 week after the inoculation of the cells until the end of the treatment period (week 6). **(b)** Average weight of NMRI nude mice orthotopically injected with PC3M-Luc2 cells and treated with vehicle (0.9% NaCl) or 2 mg/kg Foxy-5, as previously described. Mice were weighed twice per week starting the first week after inoculation of the cells until the end of the treatment period (week 6). Results are presented as the mean ± s.e.m.; and statistical significance was determined using two-way ANOVA with Bonferroni post hoc test (n.s. denotes not significant).
(TIF)

S4 Fig Effect of Foxy-5 on apoptosis *in vivo*. Relative number of Tunel-positive cells in primary tumors **(a)**, regional lymph nodes **(b)** and distal **(c)** lymph nodes of animals injected with DU145-Luc cells and treated with Foxy-5 or vehicle. Results are presented as the mean ± s.e.m.; statistical significance was determined using unpaired Student-t test (n.s. denotes not significant).
(TIFF)

S5 Fig Effect of Foxy-5 on CD44 and MMP9. **(a)** Relative number of CD44-positive cells and representative images of CD44-positive areas in primary tumors of animals injected with DU145-Luc cells and treated with Foxy-5 or vehicle. Results are presented as the mean ± s.e.m.; statistical significance was determined using unpaired Student-t test (n.s. denotes not significant). Images were taken with a 20X objective (Scale bar = 100 μm). **(b)** Secretion of MMP9 in the conditioned medium of DU145 and PC3 cells treated with Foxy-5 (100 μM) or Vehicle (0.9% NaCl) during 24 h. Results represent the mean ± s.e.m. of three (n = 3) independent experiments performed in triplicate. Statistical significance was determined using paired Student-t test with Bonferroni post hoc test (n.s. denotes not significant). **(c)** Invasion of DU145 cells pre-treated for 2 h with vehicle (0.9% NaCl) or 100 μM Foxy-5. After 2 h pre-treatment, the invasion assay was carried out over 22 h in the absence of Foxy-5. Results represent the mean ± s.e.m. of five (n = 5) independent experiments, each of which was performed in duplicate. Statistical significance was determined using paired Student-t test with Bonferroni post hoc test (*p < 0.05).
(TIFF)

S6 Fig Western blot analysis showing siRNA silencing of endogenous WNT5A in PC3 cells. Cells were transfected with either negative control siRNA (NC, 100 nM), anti-WNT5A-siRNA #1 (#1, 100 nM) or anti-WNT5A-siRNA #2 (#2, 100 nM) and incubated for 48 h. Two protein bands in the presumed WNT5A region were clearly detected in PC3 and in NC siRNA transfected cells, however only the intensity of the upper band was reduced following transfection with either WNT5A siRNA #1 or #2. A cell lysate from the WNT5A-negative human breast cancer cell line MDA-468 was used as negative control; a cell lysate from the WNT5A-positive HB2 breast cell line was used as a positive control. The lower panel shows densitometric analyses of the siRNA effects on WNT5A protein expression normalized against β-actin (n = 6).
(TIF)

S1 Table DU145-Luc metastasis incidence *in vivo*. Metastasis incidence in multiple organs in mice orthotopically injected with DU145-Luc cells and treated with either vehicle or Foxy-5. The number of organs affected in relation to all organs tested (in fraction and in percentage) is indicated.
(TIF)

S2 Table PC3M-Luc2 metastasis incidence *in vivo*. Metastasis incidence in multiple organs in mice orthotopically injected with PC3M-Luc2 cells and treated with either vehicle or Foxy-5. The number of organs affected in relation to all organs tested (in fraction and in percentage) is indicated.
(TIF)

## References

[1] Siegel R, Naishadham D, Jemal A (2013) Cancer statistics, 2013. CA Cancer J Clin 63: 11–30. doi: 10.3322/caac.21166 23335087 10.3322/caac.21166

[2] Karantanos T, Corn PG, Thompson TC (2013) Prostate cancer progression after androgen deprivation therapy: mechanisms of castrate resistance and novel therapeutic approaches. Oncogene 32: 5501–5511. doi: 10.1038/onc.2013.206 23752182 10.1038/onc.2013.206 PMC3908870

[3] Harris WP, Mostaghel EA, Nelson PS, Montgomery B (2009) Androgen deprivation therapy: progress in understanding mechanisms of resistance and optimizing androgen depletion. Nat Clin Pract Urol 6: 76–85. doi: 10.1038/ncpuro1296 19198621 10.1038/ncpuro1296 PMC2981403

[4] Nouri M, Ratther E, Stylianou N, Nelson CC, Hollier BG, Williams ED (2014) Androgen-targeted therapy-induced epithelial mesenchymal plasticity and neuroendocrine transdifferentiation in prostate cancer: an opportunity for intervention. Front Oncol 4: 370 doi: 10.3389/fonc.2014.00370 25566507 10.3389/fonc.2014.00370 PMC4274903

[5] Doctor SM, Tsao CK, Godbold JH, Galsky MD, Oh WK (2014) Is prostate cancer changing?: Evolving patterns of metastatic castration-resistant prostate cancer. Cancer 120: 833–839. doi: 10.1002/cncr.28494 25302607 10.1002/cncr.28494

[6] Quintela ML, Mateos LL, Estevez SV, Calvo OF, Herranz UA, Afonso FJ, et al (2015) Enzalutamide: A new prostate cancer targeted therapy against the androgen receptor. Cancer Treat Rev. 10.1016/j.ctrv.2014.12.006 25638257

[7] Zobniw CM, Causebrook A, Fong MK (2014) Clinical use of abiraterone in the treatment of metastatic castration-resistant prostate cancer. Res Rep Urol 6: 97–105. doi: 10.2147/RRU.S29003 25157341 10.2147/RRU.S29003 PMC4128838

[8] Martin SK, Kamelgarn M, Kyprianou N (2014) Cytoskeleton targeting value in prostate cancer treatment. Am J Clin Exp Urol 2: 15–26. 25374905 PMC4219288

[9] Ganguly SS, Li X, Miranti CK (2014) The host microenvironment influences prostate cancer invasion, systemic spread, bone colonization, and osteoblastic metastasis. Front Oncol 4: 364 doi: 10.3389/fonc.2014.00364 25566502 10.3389/fonc.2014.00364 PMC4266028

[10] Coleman RE (2006) Clinical features of metastatic bone disease and risk of skeletal morbidity. Clin Cancer Res 12: 6243s–6249s. doi: 10.1158/1078-0432.CCR-06-0931 17062708 10.1158/1078-0432.CCR-06-0931

[11] Sepulveda L, Gorgal T, Pires V, Rodrigues F (2015) Prostate cancer metastatic to the cervical lymph nodes. Case Rep Urol 2015: 263978 doi: 10.1155/2015/263978 25821627 10.1155/2015/263978 PMC4364005

[12] Zhou Y, Bolton EC, Jones JO (2015) Androgens and androgen receptor signaling in prostate tumorigenesis. J Mol Endocrinol 54: R15–R29. doi: 10.1530/JME-14-0203 25351819 10.1530/JME-14-0203

[13] Nandana S, Chung LW (2014) Prostate cancer progression and metastasis: potential regulatory pathways for therapeutic targeting. Am J Clin Exp Urol 2: 92–101. 25374910 PMC4219303

[14] Sridhar SS, Freedland SJ, Gleave ME, Higano C, Mulders P, Parker C, et al (2014) Castration-resistant prostate cancer: from new pathophysiology to new treatment. Eur Urol 65: 289–299. doi: 10.1016/j.eururo.2013.08.008 23957948 10.1016/j.eururo.2013.08.008

[15] Logan CY, Nusse R (2004) The Wnt signaling pathway in development and disease. Annu Rev Cell Dev Biol 20: 781–810. doi: 10.1146/annurev.cellbio.20.010403.113126 15473860 10.1146/annurev.cellbio.20.010403.113126

[16] Endo M, Nishita M, Fujii M, Minami Y (2015) Insight into the role of Wnt5a-induced signaling in normal and cancer cells. Int Rev Cell Mol Biol 314: 117–148. doi: 10.1016/bs.ircmb.2014.10.003 25619716 10.1016/bs.ircmb.2014.10.003

[17] Dejmek J, Dejmek A, Safholm A, Sjolander A, Andersson T (2005) Wnt-5a protein expression in primary dukes B colon cancers identifies a subgroup of patients with good prognosis. Cancer Res 65: 9142–9146. doi: 10.1158/0008-5472.CAN-05-1710 16230369 10.1158/0008-5472.CAN-05-1710

[18] Blanc E, Roux GL, Benard J, Raguenez G (2005) Low expression of Wnt-5a gene is associated with high-risk neuroblastoma. Oncogene 24: 1277–1283. doi: 10.1038/sj.onc.1208255 15592517 10.1038/sj.onc.1208255

[19] Jonsson M, Dejmek J, Bendahl PO, Andersson T (2002) Loss of Wnt-5a protein is associated with early relapse in invasive ductal breast carcinomas. Cancer Res 62: 409–416. 11809689

[20] Liang H, Chen Q, Coles AH, Anderson SJ, Pihan G, Bradley A, et al (2003) Wnt5a inhibits B cell proliferation and functions as a tumor suppressor in hematopoietic tissue. Cancer Cell 4: 349–360. 14667502 10.1016/s1535-6108(03)00268-x

[21] Kurayoshi M, Oue N, Yamamoto H, Kishida M, Inoue A, Asahara T, et al (2006) Expression of Wnt-5a is correlated with aggressiveness of gastric cancer by stimulating cell migration and invasion. Cancer Res 66: 10439–10448. doi: 10.1158/0008-5472.CAN-06-2359 17079465 10.1158/0008-5472.CAN-06-2359

[22] Da Forno PD, Pringle JH, Hutchinson P, Osborn J, Huang Q, Potter L, et al (2008) WNT5A expression increases during melanoma progression and correlates with outcome. Clin Cancer Res 14: 5825–5832. doi: 10.1158/1078-0432.CCR-07-5104 18794093 10.1158/1078-0432.CCR-07-5104

[23] Huang CL, Liu D, Nakano J, Ishikawa S, Kontani K, Yokomise H, et al (2005) Wnt5a expression is associated with the tumor proliferation and the stromal vascular endothelial growth factor—an expression in non-small-cell lung cancer. J Clin Oncol 23: 8765–8773. doi: 10.1200/JCO.2005.02.2871 16314637 10.1200/JCO.2005.02.2871

[24] Ripka S, Konig A, Buchholz M, Wagner M, Sipos B, Kloppel G, et al (2007) WNT5A—target of CUTL1 and potent modulator of tumor cell migration and invasion in pancreatic cancer. Carcinogenesis 28: 1178–1187. doi: 10.1093/carcin/bgl255 17227781 10.1093/carcin/bgl255

[25] Syed Khaja AS, Helczynski L, Edsjo A, Ehrnstrom R, Lindgren A, Ulmert D, et al (2011) Elevated level of Wnt5a protein in localized prostate cancer tissue is associated with better outcome. PLoS One 6: e26539 doi: 10.1371/journal.pone.0026539 22039506 10.1371/journal.pone.0026539 PMC3200334

[26] Khaja AS, Egevad L, Helczynski L, Wiklund P, Andersson T, Bjartell A (2012) Emphasizing the role of Wnt5a protein expression to predict favorable outcome after radical prostatectomy in patients with low-grade prostate cancer. Cancer Med 1: 96–104. doi: 10.1002/cam4.5 23342259 10.1002/cam4.5 PMC3544436

[27] Yamamoto H, Oue N, Sato A, Hasegawa Y, Yamamoto H, Matsubara A, et al (2010) Wnt5a signaling is involved in the aggressiveness of prostate cancer and expression of metalloproteinase. Oncogene 29: 2036–2046. doi: 10.1038/onc.2009.496 20101234 10.1038/onc.2009.496

[28] Thiele S, Gobel A, Rachner TD, Fuessel S, Froehner M, Muders MH, et al (2014) WNT5A has Anti-Prostate Cancer Effects In Vitro and Reduces Tumor Growth in the Skeleton In Vivo. J Bone Miner Res. 10.1002/jbmr.2362 25224731

[29] Sandsmark E, Hansen AF, Selnaes KM, Bertilsson H, Bofin AM, Wright AJ, et al (2016) A novel non-canonical Wnt signature for prostate cancer aggressiveness. Oncotarget. 10.18632/oncotarget.14161 PMC5354754 28030815

[30] Miyamoto DT, Zheng Y, Wittner BS, Lee RJ, Zhu H, Broderick KT, et al (2015) RNA-Seq of single prostate CTCs implicates noncanonical Wnt signaling in antiandrogen resistance. Science 349: 1351–1356. doi: 10.1126/science.aab0917 26383955 10.1126/science.aab0917 PMC4872391

[31] Safholm A, Leandersson K, Dejmek J, Nielsen CK, Villoutreix BO, Andersson T (2006) A formylated hexapeptide ligand mimics the ability of Wnt-5a to impair migration of human breast epithelial cells. J Biol Chem 281: 2740–2749. doi: 10.1074/jbc.M508386200 16330545 10.1074/jbc.M508386200

[32] Safholm A, Tuomela J, Rosenkvist J, Dejmek J, Harkonen P, Andersson T (2008) The Wnt-5a-derived hexapeptide Foxy-5 inhibits breast cancer metastasis in vivo by targeting cell motility. Clin Cancer Res 14: 6556–6563. doi: 10.1158/1078-0432.CCR-08-0711 18927296 10.1158/1078-0432.CCR-08-0711

[33] Canesin G, Evans-Axelsson S, Hellsten R, Sterner O, Krzyzanowska A, Andersson T, et al (2015) The STAT3 Inhibitor Galiellalactone Effectively Reduces Tumor Growth and Metastatic Spread in an Orthotopic Xenograft Mouse Model of Prostate Cancer. Eur Urol. 10.1016/j.eururo.2015.06.016 26144873

[34] Canesin G, Cuevas EP, Santos V, Lopez-Menendez C, Moreno-Bueno G, Huang Y, et al (2014) Lysyl oxidase-like 2 (LOXL2) and E47 EMT factor: novel partners in E-cadherin repression and early metastasis colonization. Oncogene 0. 10.1038/onc.2014.23 24632622

[35] Linnskog R, Jonsson G, Axelsson L, Prasad CP, Andersson T (2014) Interleukin-6 drives melanoma cell motility through p38alpha-MAPK-dependent up-regulation of WNT5A expression. Mol Oncol. 10.1016/j.molonc.2014.05.008 PMC5528610 24954857

[36] Hellsten R, Johansson M, Dahlman A, Dizeyi N, Sterner O, Bjartell A (2008) Galiellalactone is a novel therapeutic candidate against hormone-refractory prostate cancer expressing activated Stat3. Prostate 68: 269–280. doi: 10.1002/pros.20699 18163422 10.1002/pros.20699

[37] Jantscheff P, Esser N, Graeser R, Ziroli V, Kluth J, Unger C, et al (2009) Liposomal gemcitabine (GemLip)-efficient drug against hormone-refractory Du145 and PC-3 prostate cancer xenografts. Prostate 69: 1151–1163. doi: 10.1002/pros.20964 19399788 10.1002/pros.20964

[38] Jiang W, Crossman DK, Mitchell EH, Sohn P, Crowley MR, Serra R (2013) WNT5A inhibits metastasis and alters splicing of Cd44 in breast cancer cells. PLoS One 8: e58329 doi: 10.1371/journal.pone.0058329 23484019 10.1371/journal.pone.0058329 PMC3590134

[39] Prasad CP, Chaurasiya SK, Guilmain W, Andersson T (2016) WNT5A signaling impairs breast cancer cell migration and invasion via mechanisms independent of the epithelial-mesenchymal transition. J Exp Clin Cancer Res 35: 144 doi: 10.1186/s13046-016-0421-0 27623766 10.1186/s13046-016-0421-0 PMC5022188

[40] Prasad CP, Chaurasiya SK, Axelsson L, Andersson T (2013) WNT-5A triggers Cdc42 activation leading to an ERK1/2 dependent decrease in MMP9 activity and invasive migration of breast cancer cells. Mol Oncol 7: 870–883. doi: 10.1016/j.molonc.2013.04.005 23727359 10.1016/j.molonc.2013.04.005 PMC5528454

[41] Chen C, Zhang Q, Liu S, Parajuli KR, Qu Y, Mei J, et al (2015) IL-17 and insulin/IGF1 enhance adhesion of prostate cancer cells to vascular endothelial cells through CD44-VCAM-1 interaction. Prostate 75: 883–895. doi: 10.1002/pros.22971 25683512 10.1002/pros.22971 PMC4405436

[42] Peng X, Zhou Y, Tian H, Yang G, Li C, Geng Y, et al (2015) Sulforaphane inhibits invasion by phosphorylating ERK1/2 to regulate E-cadherin and CD44v6 in human prostate cancer DU145 cells. Oncol Rep 34: 1565–1572. doi: 10.3892/or.2015.4098 26134113 10.3892/or.2015.4098

[43] Zhang C, Shu L, Kim H, Khor TO, Wu R, Li W, et al (2016) Phenethyl isothiocyanate (PEITC) suppresses prostate cancer cell invasion epigenetically through regulating microRNA-194. Mol Nutr Food Res. 10.1002/mnfr.201500918 PMC5495185 26820911

[44] Wu J, Ji A, Wang X, Zhu Y, Yu Y, Lin Y, et al (2015) MicroRNA-195-5p, a new regulator of Fra-1, suppresses the migration and invasion of prostate cancer cells. J Transl Med 13: 289 doi: 10.1186/s12967-015-0650-6 26337460 10.1186/s12967-015-0650-6 PMC4558968

[45] Reel B, Korkmaz CG, Arun MZ, Yildirim G, Ogut D, Kaymak A, et al (2015) The Regulation of Matrix Metalloproteinase Expression and the Role of Discoidin Domain Receptor 1/2 Signalling in Zoledronate-treated PC3 Cells. J Cancer 6: 1020–1029. doi: 10.7150/jca.12733 26366216 10.7150/jca.12733 PMC4565852

[46] Tseng JC, Lin CY, Su LC, Fu HH, Yang SD, Chuu CP (2016) CAPE suppresses migration and invasion of prostate cancer cells via activation of non-canonical Wnt signaling. Oncotarget. 10.18632/oncotarget.9380 PMC5122368 27191743

[47] Asem MS, Buechler S, Wates RB, Miller DL, Stack MS (2016) Wnt5a Signaling in Cancer. Cancers (Basel) 8. 10.3390/cancers8090079 PMC5040981 27571105

[48] Medrek C, Landberg G, Andersson T, Leandersson K (2009) Wnt-5a-CKI{alpha} signaling promotes {beta}-catenin/E-cadherin complex formation and intercellular adhesion in human breast epithelial cells. J Biol Chem 284: 10968–10979. doi: 10.1074/jbc.M804923200 19244247 10.1074/jbc.M804923200 PMC2667782

[49] Jonsson M, Andersson T (2001) Repression of Wnt-5a impairs DDR1 phosphorylation and modifies adhesion and migration of mammary cells. J Cell Sci 114: 2043–2053. 11493640 10.1242/jcs.114.11.2043

[50] Schulte G (2010) International Union of Basic and Clinical Pharmacology. LXXX. The class Frizzled receptors. Pharmacol Rev 62: 632–667. doi: 10.1124/pr.110.002931 21079039 10.1124/pr.110.002931

[51] Nusse R, Fuerer C, Ching W, Harnish K, Logan C, Zeng A, et al (2008) Wnt signaling and stem cell control. Cold Spring Harb Symp Quant Biol 73: 59–66. doi: 10.1101/sqb.2008.73.035 19028988 10.1101/sqb.2008.73.035

[52] Liss MA, Schlicht M, Kahler A, Fitzgerald R, Thomassi T, Degueme A, et al (2010) Characterization of soy-based changes in Wnt-frizzled signaling in prostate cancer. Cancer Genomics Proteomics 7: 245–252. 20952759

[53] Ren W, Li C, Duan W, Du S, Yang F, Zhou J, et al (2016) MicroRNA-613 represses prostate cancer cell proliferation and invasion through targeting Frizzled7. Biochem Biophys Res Commun 469: 633–638. doi: 10.1016/j.bbrc.2015.12.054 26703210 10.1016/j.bbrc.2015.12.054

[54] Ma F, Ye H, He HH, Gerrin SJ, Chen S, Tanenbaum BA, et al (2016) SOX9 drives WNT pathway activation in prostate cancer. J Clin Invest 126: 1745–1758. doi: 10.1172/JCI78815 27043282 10.1172/JCI78815 PMC4855922

[55] Thiele S, Rauner M, Goettsch C, Rachner TD, Benad P, Fuessel S, et al (2011) Expression profile of WNT molecules in prostate cancer and its regulation by aminobisphosphonates. J Cell Biochem 112: 1593–1600. doi: 10.1002/jcb.23070 21344486 10.1002/jcb.23070

[56] Bennett LL, Ingason A (2014) Enzalutamide (Xtandi) for patients with metastatic, resistant prostate cancer. Ann Pharmacother 48: 530–537. doi: 10.1177/1060028013518899 24458946 10.1177/1060028013518899

[57] van Dodewaard-de Jong JM, Verheul HM, Bloemendal HJ, de Klerk JM, Carducci MA, van den Eertwegh AJ (2015) New Treatment Options for Patients With Metastatic Prostate Cancer: What Is The Optimal Sequence? Clin Genitourin Cancer. 10.1016/j.clgc.2015.01.008 25704270

[58] Mouraviev V, Mariados N, Albala D, Concepcion RS, Shore ND, Sims RB, et al (2014) The Rationale for Optimal Combination Therapy With Sipuleucel-T for Patients With Castration-resistant Prostate Cancer. Rev Urol 16: 122–130. 25337042 PMC4191632

