# Supplementary figures and images for: Treatment with the WNT5A-mimicking peptide Foxy-5 effectively reduces the metastatic spread of WNT5A-low prostate cancer cells in an orthotopic mouse model
Source: PLoS One. 2017 Sep 8;12(9):e0184418. doi: 10.1371/journal.pone.0184418 (PMC5590932; doi:10.1371/journal.pone.0184418)

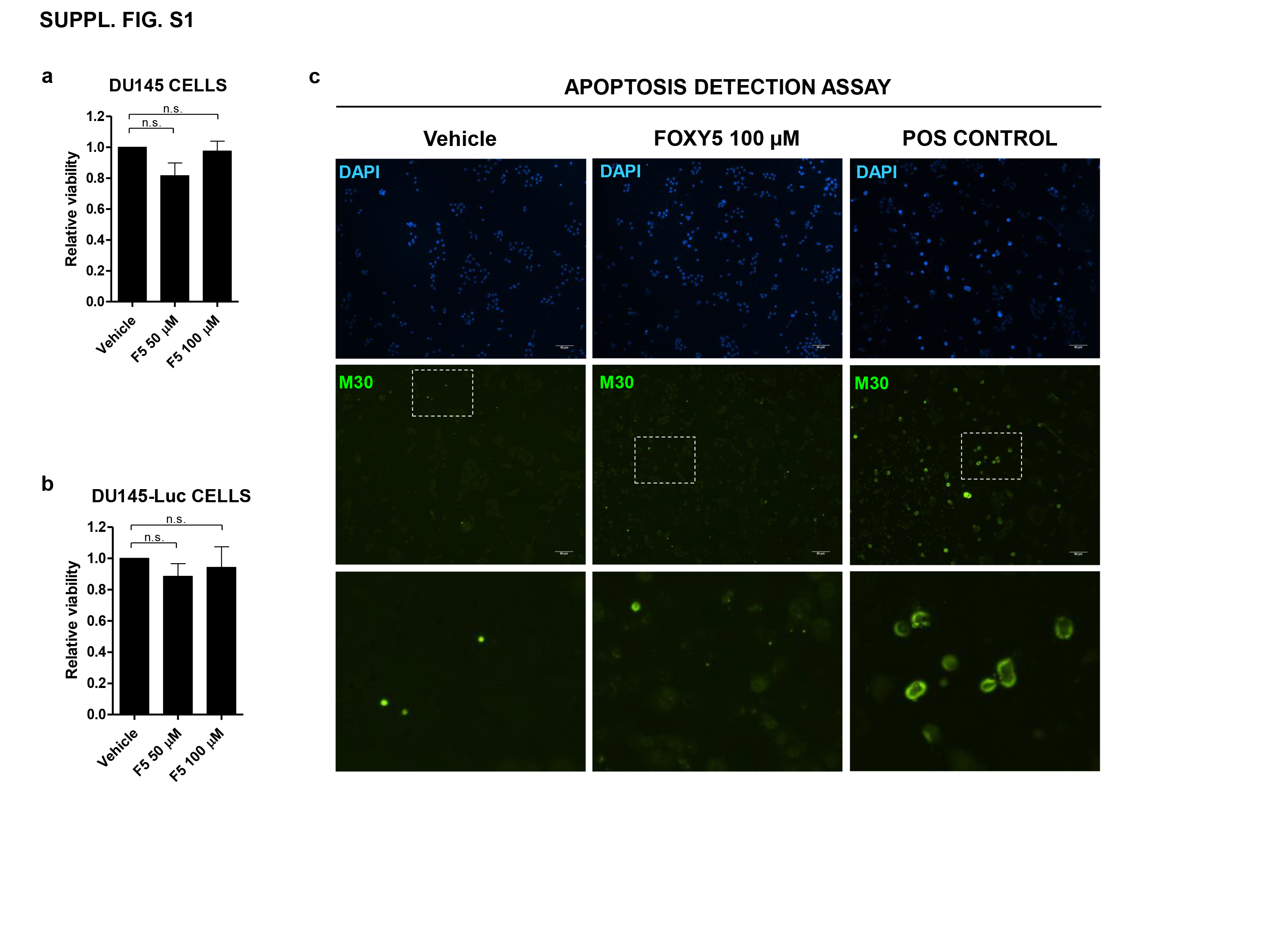

Supplement: S1 Fig — (a-b) MTT viability assays on DU145 (a) and DU145-Luc cells (b) after 24 h of treatment with vehicle (0.9% NaCl) or 50/100 μM Foxy-5. Results represent the mean ± s.e.m. of seven (n = 7) and eleven (n = 11) independent experiments, respectively, each of which was performed in quadruplicate. Statistical significance was determined using one-way ANOVA with Bonferroni post hoc test (n.s. denotes not significant). (c) Immunofluorescence detection of apoptotic DU145-Luc cells after 24 h of treatment with vehicle (left panels), 100 μM Foxy-5 (middle panels) or 5 μM of the apoptosis inducing compound Galiellalactone (right panels). Apoptotic cells were visualized with the M30-cytodeath antibody, and all nuclei were stained with DAPI. Images were taken with a 10X objective (Scale bar = 50 μm). The indicated areas in the middle panels are magnified and shown in the three lower panels. (TIF) [file pone.0184418.s001.tif]

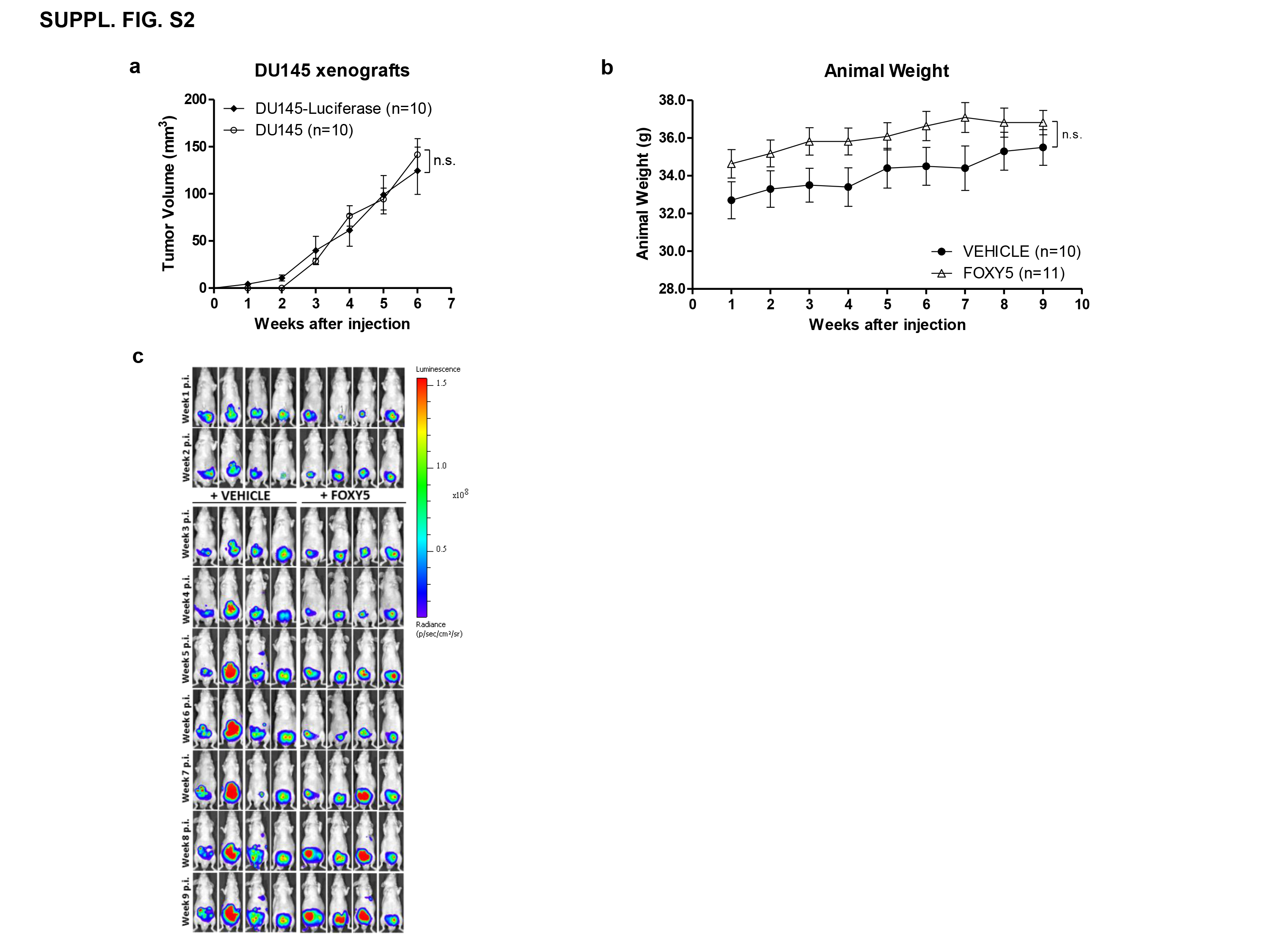

Supplement: S2 Fig — (a) A comparison of tumor growth between DU145 and DU145-Luc cells injected subcutaneously into the flanks of NMRI nude mice (n = 10 per group). Tumor size was measured weekly with a caliper, and the volume was calculated as previously described [36]. Results are presented as the mean ± s.e.m.; and statistical significance was determined using two-way ANOVA with Bonferroni post hoc test (n.s. denotes not significant). (b) Average weight of NMRI nude mice orthotopically injected with DU145-Luc cells and treated with vehicle (0.9% NaCl) or 2 mg/kg Foxy-5, as previously described. Mice were weighed twice per week starting the first week after inoculation of the cells until the end of the treatment period (week 9). Results are presented as the mean ± s.e.m.; and statistical significance was determined using two-way ANOVA with Bonferroni post hoc test (n.s. denotes not significant). (c) Representative bioluminescence images of NMRI nude mice orthotopically injected with DU145-Luc cells and treated via intraperitoneal injections with either vehicle (NaCl 0.9%) or Foxy-5 (2 mg/kg in 0.9% NaCl) every other day between weeks 3 and 9. Images were taken weekly starting 1 week after the inoculation of the cells until the end of the treatment period. (TIF) [file pone.0184418.s002.tif]

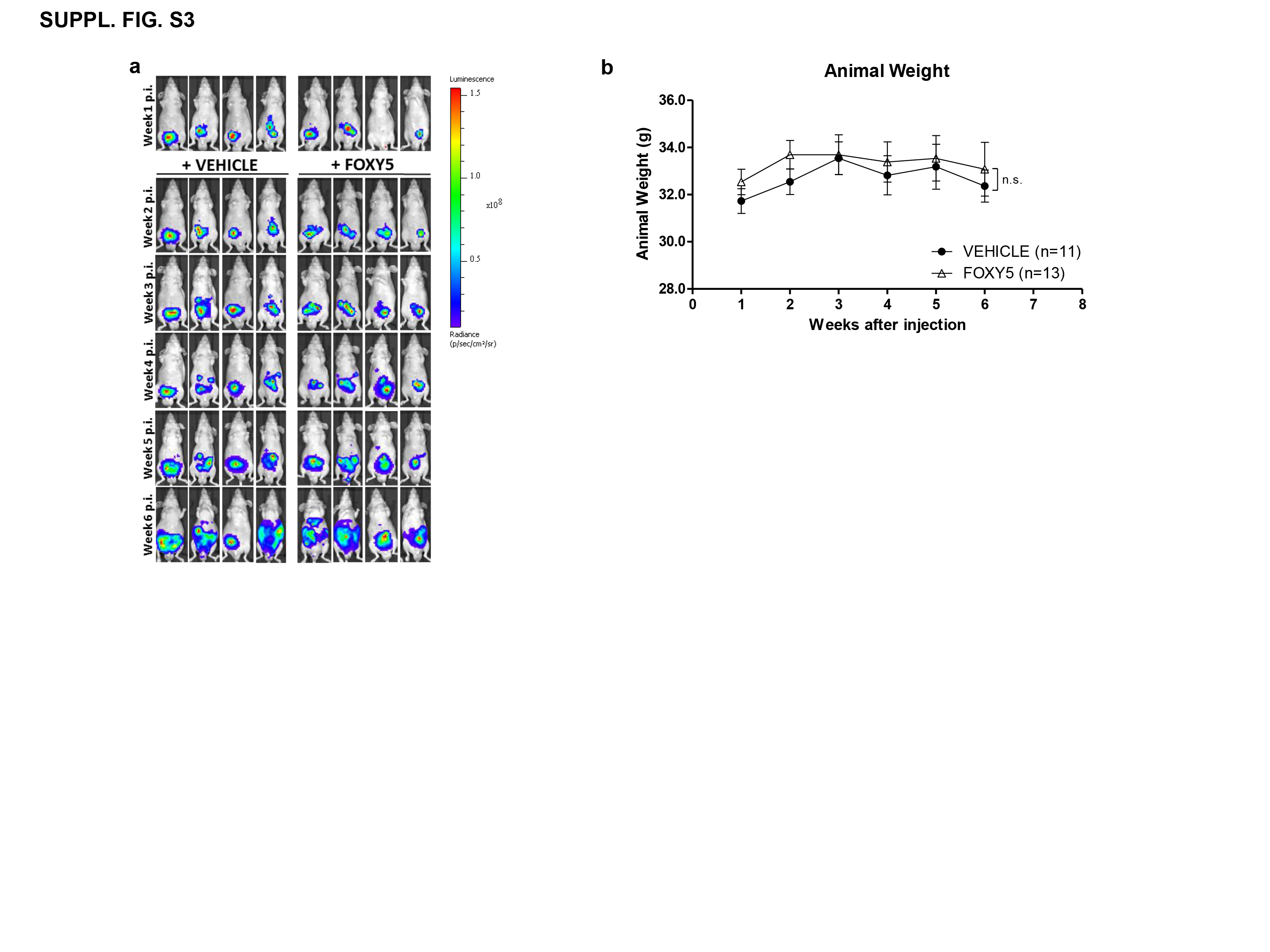

Supplement: S3 Fig — (a) Representative bioluminescence images of NMRI nude mice orthotopically injected with PC3M-Luc2 cells and treated via intraperitoneal injections with either vehicle (NaCl 0.9%) or Foxy-5 (2 mg/kg in 0.9% NaCl) every other day between weeks 2 and 6. Images were taken weekly starting 1 week after the inoculation of the cells until the end of the treatment period (week 6). (b) Average weight of NMRI nude mice orthotopically injected with PC3M-Luc2 cells and treated with vehicle (0.9% NaCl) or 2 mg/kg Foxy-5, as previously described. Mice were weighed twice per week starting the first week after inoculation of the cells until the end of the treatment period (week 6). Results are presented as the mean ± s.e.m.; and statistical significance was determined using two-way ANOVA with Bonferroni post hoc test (n.s. denotes not significant). (TIF) [file pone.0184418.s003.tif]

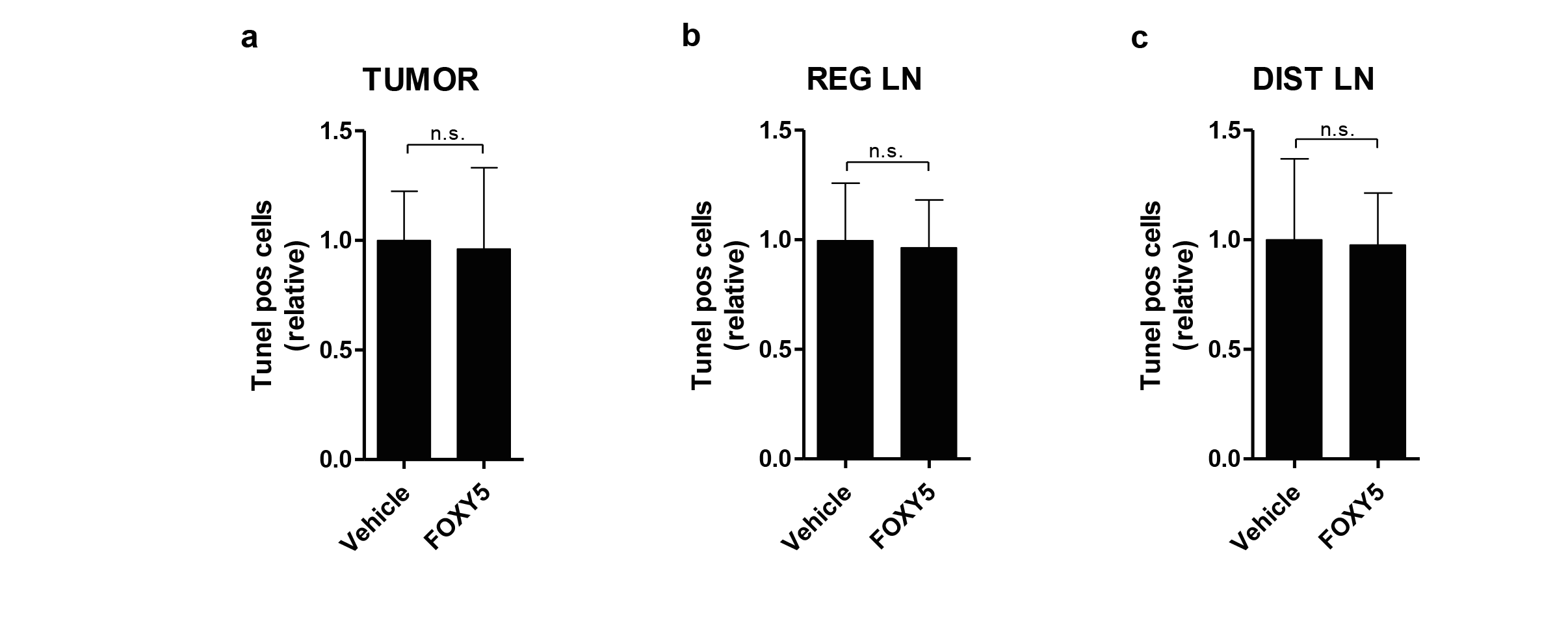

Supplement: S4 Fig — Relative number of Tunel-positive cells in primary tumors (a), regional lymph nodes (b) and distal (c) lymph nodes of animals injected with DU145-Luc cells and treated with Foxy-5 or vehicle. Results are presented as the mean ± s.e.m.; statistical significance was determined using unpaired Student-t test (n.s. denotes not significant). (TIFF) [file pone.0184418.s004.tiff]

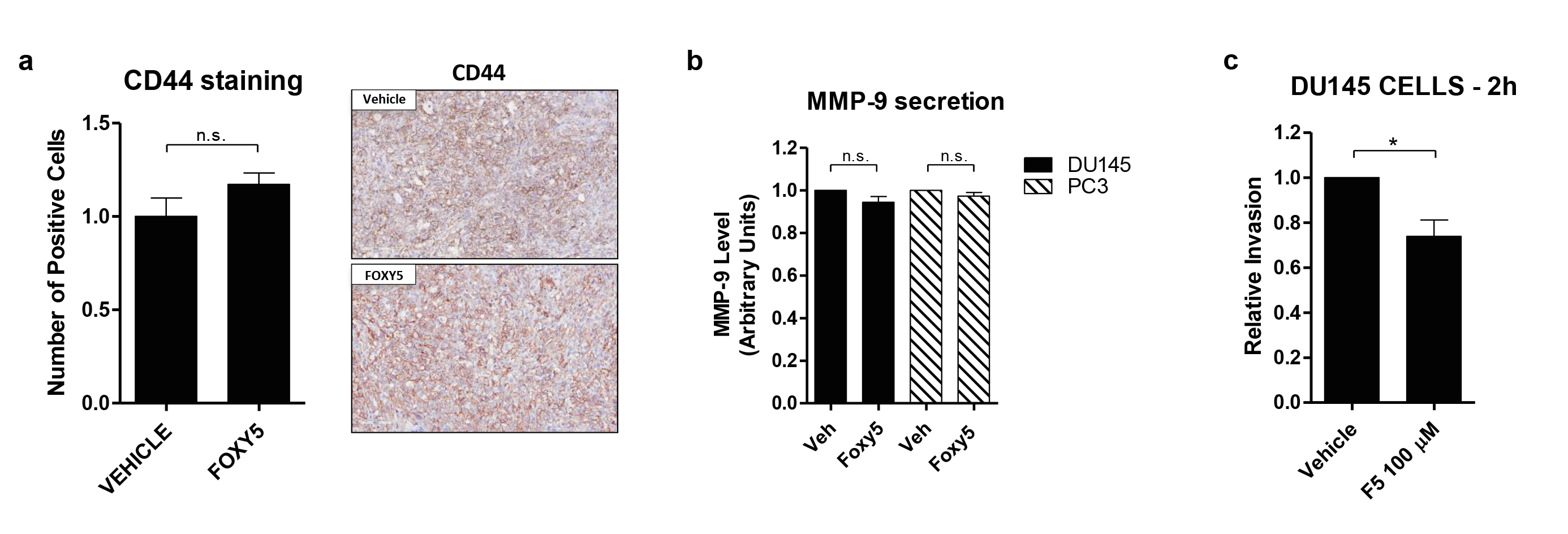

Supplement: S5 Fig — (a) Relative number of CD44-positive cells and representative images of CD44-positive areas in primary tumors of animals injected with DU145-Luc cells and treated with Foxy-5 or vehicle. Results are presented as the mean ± s.e.m.; statistical significance was determined using unpaired Student-t test (n.s. denotes not significant). Images were taken with a 20X objective (Scale bar = 100 μm). (b) Secretion of MMP9 in the conditioned medium of DU145 and PC3 cells treated with Foxy-5 (100 μM) or Vehicle (0.9% NaCl) during 24 h. Results represent the mean ± s.e.m. of three (n = 3) independent experiments performed in triplicate. Statistical significance was determined using paired Student-t test with Bonferroni post hoc test (n.s. denotes not significant). (c) Invasion of DU145 cells pre-treated for 2 h with vehicle (0.9% NaCl) or 100 μM Foxy-5. After 2 h pre-treatment, the invasion assay was carried out over 22 h in the absence of Foxy-5. Results represent the mean ± s.e.m. of five (n = 5) independent experiments, each of which was performed in duplicate. Statistical significance was determined using paired Student-t test with Bonferroni post hoc test (*p < 0.05). (TIFF) [file pone.0184418.s005.tiff]

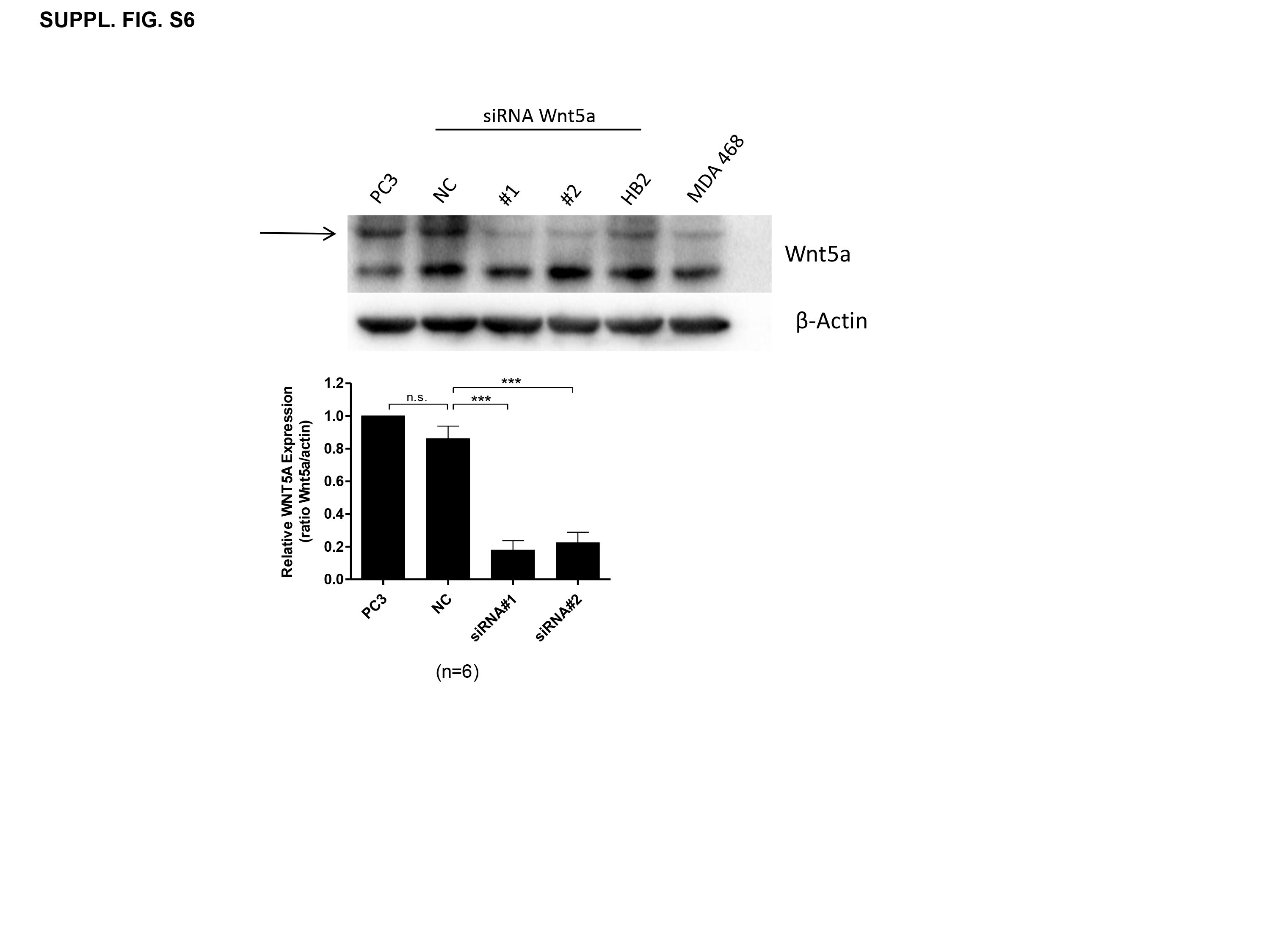

Supplement: S6 Fig — Cells were transfected with either negative control siRNA (NC, 100 nM), anti-WNT5A-siRNA #1 (#1, 100 nM) or anti-WNT5A-siRNA #2 (#2, 100 nM) and incubated for 48 h. Two protein bands in the presumed WNT5A region were clearly detected in PC3 and in NC siRNA transfected cells, however only the intensity of the upper band was reduced following transfection with either WNT5A siRNA #1 or #2. A cell lysate from the WNT5A-negative human breast cancer cell line MDA-468 was used as negative control; a cell lysate from the WNT5A-positive HB2 breast cell line was used as a positive control. The lower panel shows densitometric analyses of the siRNA effects on WNT5A protein expression normalized against β-actin (n = 6). (TIF) [file pone.0184418.s006.tif]

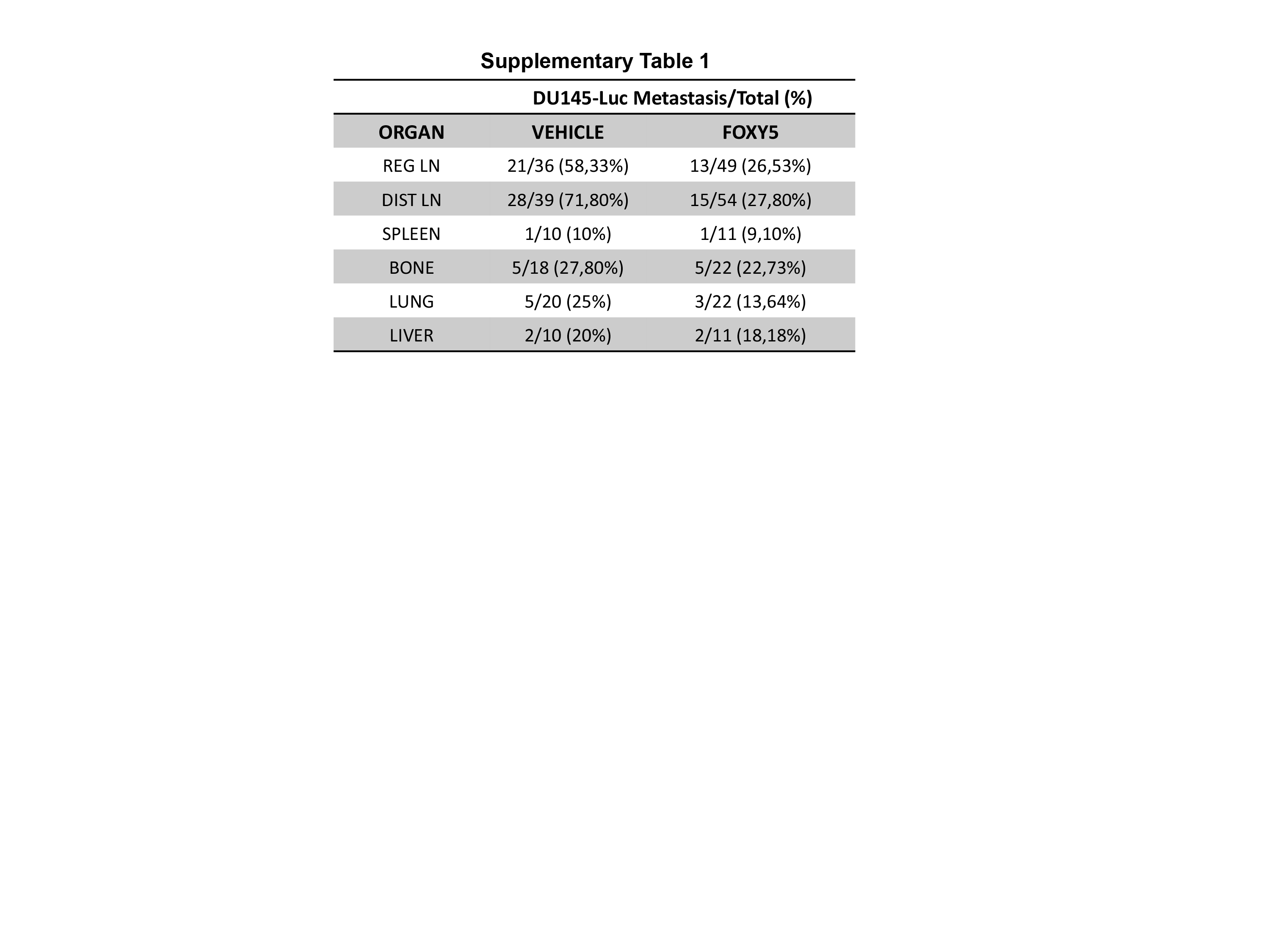

Supplement: S1 Table — Metastasis incidence in multiple organs in mice orthotopically injected with DU145-Luc cells and treated with either vehicle or Foxy-5. The number of organs affected in relation to all organs tested (in fraction and in percentage) is indicated. (TIF) [file pone.0184418.s007.tif]

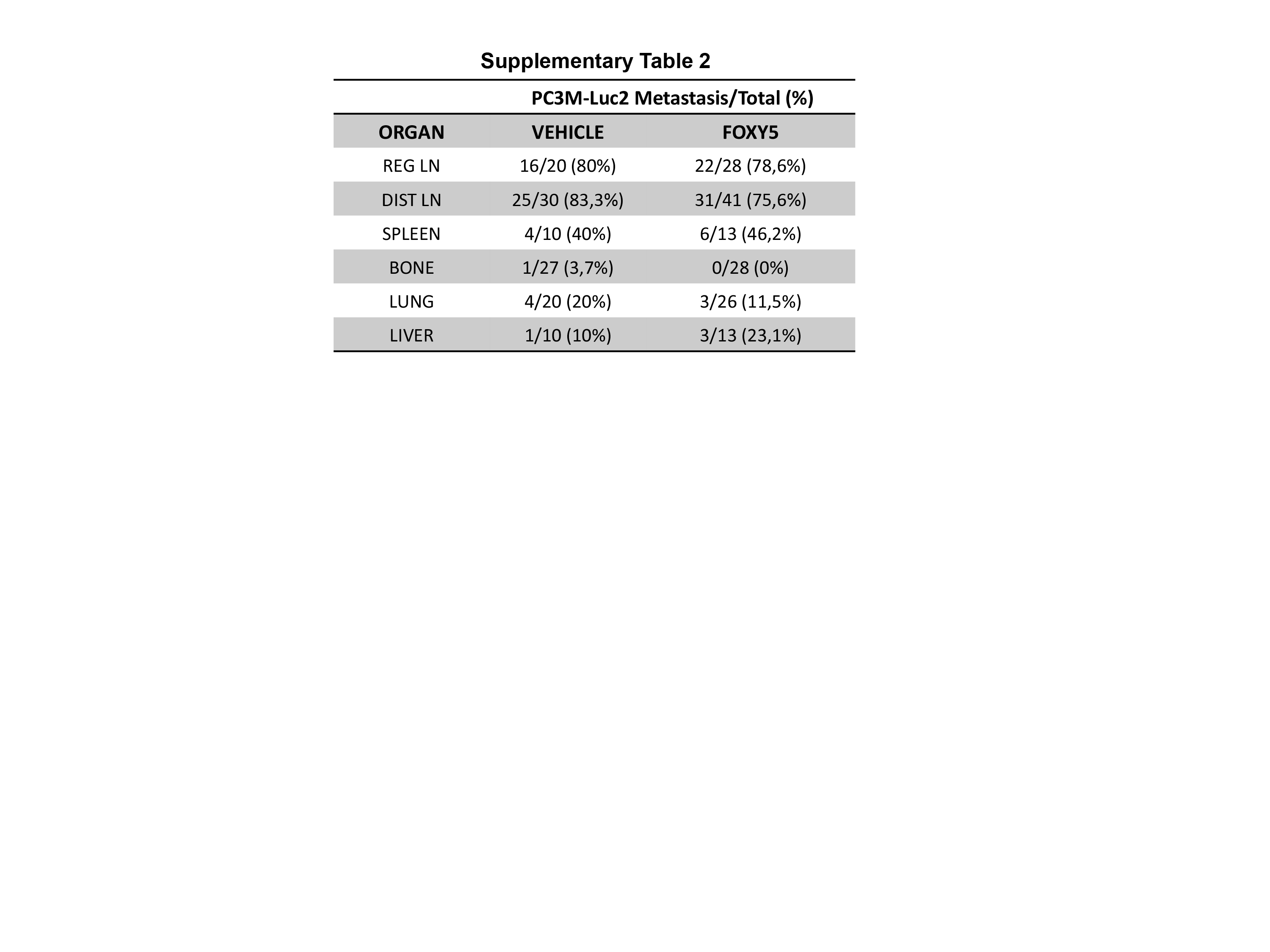

Supplement: S2 Table — Metastasis incidence in multiple organs in mice orthotopically injected with PC3M-Luc2 cells and treated with either vehicle or Foxy-5. The number of organs affected in relation to all organs tested (in fraction and in percentage) is indicated. (TIF) [file pone.0184418.s008.tif]
